# Directed Evolution Improves the Catalytic Efficiency of APEX2‐Mediated Proximity‐Dependent RNA Labeling

**DOI:** 10.1002/advs.75012

**Published:** 2026-03-28

**Authors:** Gang Wang, Yi Li, Peiyuan Meng, Peng Zou

**Affiliations:** ^1^ College of Chemistry and Molecular Engineering Synthetic and Functional Biomolecules Center Beijing National Laboratory For Molecular Sciences PKU‐IDG/McGovern Institute for Brain Research Key Laboratory of Bioorganic Chemistry and Molecular Engineering of Ministry of Education Beijing Advanced Center of RNA Biology (BEACON), Peking University Beijing China; ^2^ Academy for Advanced Interdisciplinary Studies PKU‐Tsinghua Center for Life Science Peking University Beijing China; ^3^ Chinese Institute For Brain Research (CIBR) Beijing China

## Abstract

Engineered ascorbate peroxidase APEX2 has been widely used for spatially restricted profiling of subcellular biomolecules, but its catalytic efficiency toward newly developed probes such as biotin‐aniline (Btn‐An) remains suboptimal. To overcome this limitation, we performed yeast surface display‐based directed evolution to enhance APEX2 activity toward Btn‐An. The resulting variant, ^L242F^APEX2, exhibits an approximately two‐fold improvement in labeling efficiency, likely through enhanced enzyme‐substrate interactions. This increased activity enables rapid and efficient proximity labeling while maintaining spatial specificity. After validating its performance at the ER membrane, we applied ^L242F^APEX2 to profile the transcriptome proximal to the midbody and identified *ANLN* as a previously unreported midbody‐localized mRNA during telophase. Drug perturbation and reporter assays further revealed that *ANLN* mRNA targeting to the midbody occurs co‐translationally and depends on its nascent N‐terminal peptide. Together, this work establishes ^L242F^APEX2 as an improved and versatile tool for spatially resolved transcriptomics in complex subcellular contexts.

## 1 Introduction

**FIGURE 1:**
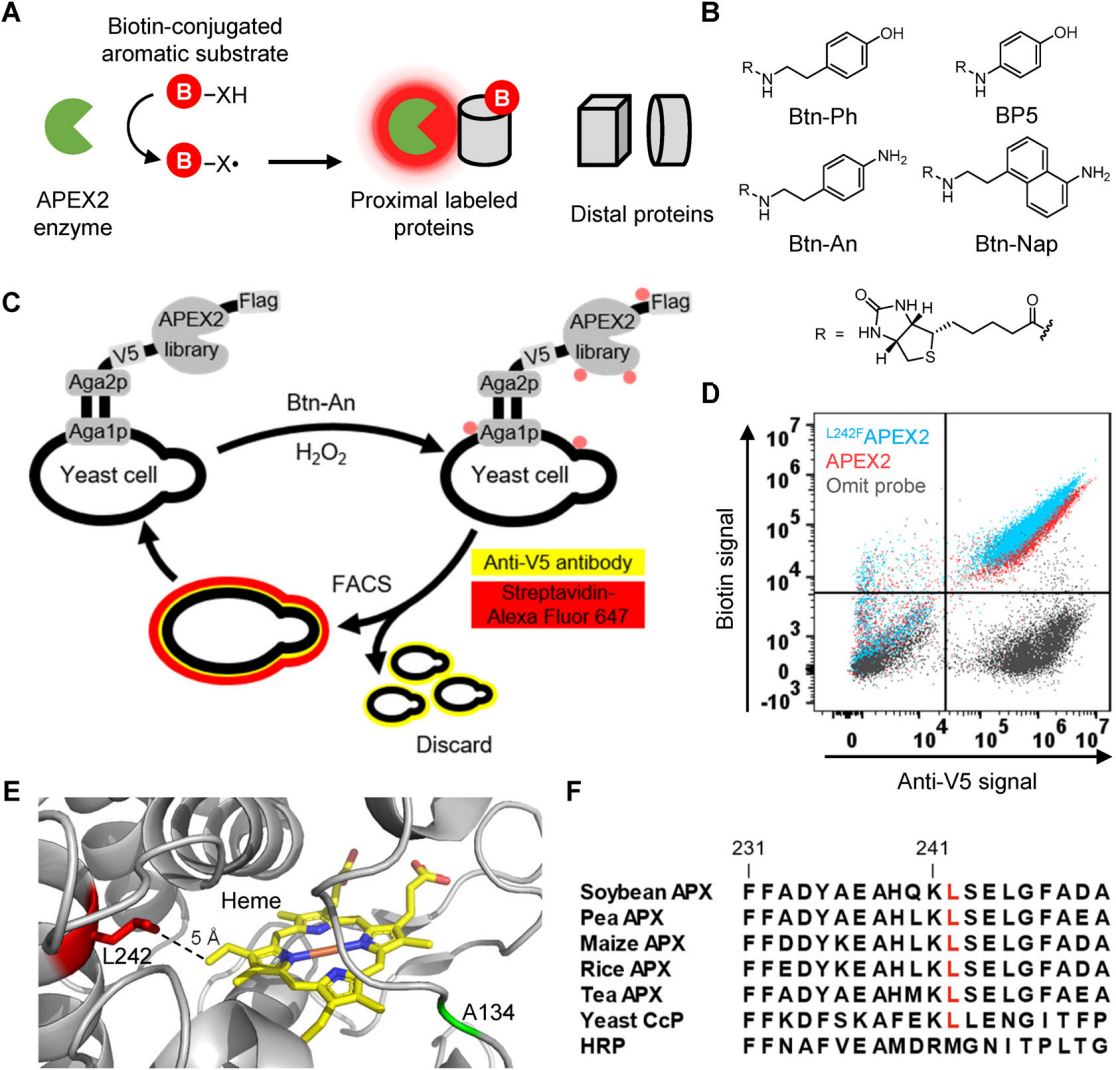
Directed evolution on APEX2, identifying ^L242F^APEX2 mutant with higher Btn‐An labeling activity. (A) Scheme showing APEX2‐based proximity‐dependent proteome labeling. (B) Chemical structures of the active aromatic substrates used in this work. (C) Scheme of yeast surface display‐based APEX2 evolution with the Btn‐An probe. (D) FACS analysis comparing the Btn‐An labeling intensity for APEX2 variants. (E) A zoom‐in view of soybean APX structure (PDB ID: 1V0H) with mutation sites in APEX2 and ^L242F^APEX2. (F) Sequence homology comparison of APX in various species, yeast CcP, and HRP.

Understanding the molecular mechanism and bio‐macromolecular inventory in specific subcellular compartments is essential for comprehending how cellular processes are regulated. Engineered ascorbate peroxidases, such as APEX, have emerged as powerful tools for mapping the subcellular proteome with high temporal and spatial resolution [1, 2]. When fused with a protein of interest, APEX catalyzes the oxidation of biotin‐phenol (Btn‐Ph) substrate to phenoxyl radicals in the presence of hydrogen peroxide (H_2_O_2_). These active phenoxyl radicals then form covalent bonds with adjacent electron‐rich amino acids, primarily tyrosine, as well as tryptophan and cysteine [1, 3]. Due to their limited diffusion radius (∼200 nm), phenoxyl radicals predominantly label proteins that are in direct contact with or in close proximity to the APEX enzyme [4, 5] (Figure 1A). To enhance the labeling intensity of APEX, the Ting lab has developed APEX2 (APEX A134P mutation) via directed evolution on a yeast surface display platform [6]. This mutation exhibits increased labeling activity even at low expression levels. Additionally, a cysteine‐free single mutant, C32S of APEX2, has been reported to show more stable expression when fused to multi‐pass membrane proteins [7].

In addition to evolving the APEX enzyme itself, innovations in labeling substrates have expanded the applications of the APEX method. In previous research, our laboratory screened a panel of aromatic APEX2 substrates and identified biotin‐aniline (Btn‐An) and biotin‐naphthylamine (Btn‐Nap) probes with increased reactivity toward RNA and DNA, respectively [8]. These probes demonstrated higher bond dissociation energy compared to the Btn‐Ph probe, suggesting their ability to oxidize guanine nucleobase, which possesses the lowest redox potential among the five nucleobases [9]. More recently, Tian and co‐workers utilized another active phenol substrate, BP5, which exhibited higher spatial resolution in mapping the interactome of EGFR signaling component STS1 [10].

These active probes offer two key advantages for proteomic or transcriptomic mapping. First, higher catalytic oxidation capacity enables labeling of a wider range of substrate species. The high‐energy radicals formed can covalently bond with amino acid residues or nucleobases. Second, the active radicals are susceptible to quenching by solvent, endogenous biomolecules, or themselves during diffusion, resulting in higher labeling specificity. It is evident that the employment of active APEX probes would advance the APEX method with improved spatiotemporal resolution and broader application prospects. However, APEX2 was initially engineered for improved catalytic efficiency toward the Btn‐Ph substrate, rather than these newly developed probes.

Herein, we utilized yeast surface display‐based directed evolution to enhance APEX2 activity toward the high‐activity probe Btn‐An. This effort yielded the APEX2 L242F variant (hereafter denoted as ^L242F^APEX2), which exhibited an approximately two‐fold increase in labeling intensity. This variant likely enhances the production of aniline radicals through more effective interaction with the Btn‐An probe. When combined with 5 mM Btn‐An substrate, ^L242F^APEX2 offers substantially boosted labeling efficiency without compromising spatial specificity, thus allowing a notable reduction in the labeling time window. After validating the spatial specificity in the ER membrane, we applied ^L242F^APEX2 to profile the midbody‐proximal transcriptome and identified *ANLN* as a novel midbody‐localized transcript during telophase. Finally, through drug perturbation and reporter mRNA assays, we demonstrated that the midbody targeting of *ANLN* mRNA occurs via a co‐translational mechanism mediated by its nascent N‐terminal polypeptide.

## 2 Results

### 2.1 Directed Evolution of APEX2 for Higher Labeling Intensity with Btn‐An Probe

Prior to embarking on directed evolution, our aim is to characterize the labeling activity of several APEX probes in mammalian cells or on the yeast surface. This initial step will enable us to select the most suitable probe for subsequent labeling and screening processes (Figure 1B). In HEK293T cells expressing mitochondria‐localized APEX2 (mito‐APEX2), the Btn‐Ph probe exhibited significantly higher labeling intensity compared to other probes (Figure S1A). Conversely, in HEK293T cells expressing endoplasmic reticulum (ER) lumen localized horse radish peroxidase (ER‐HRP), both the Btn‐Ph and Btn‐An probes demonstrated comparable labeling intensity (Figure S1A). We speculate from the above data that APEX2 might not be as suitable for Btn‐An labeling, given that it was initially screened under Btn‐Ph labeling on the yeast surface. Additionally, the distinct Western blot (WB) labeling patterns of Btn‐Ph and Btn‐An probes suggest differing protein selectivity between them. When catalyzed by APEX2 anchored on the yeast surface, the Btn‐Ph probe exhibited the highest labeling intensity, followed by the BP5 probe and then the Btn‐An probe (Figure S1B). The labeling signal of Btn‐Nap could not be detected by fluorescence‐activated cell sorting (FACS) on the yeast surface. Therefore, we have opted to focus on further APEX2 engineering using the Btn‐An and BP5 probes.

A random mutant library of 2 × 10^7^ APEX2 variants was generated through an error‐prone PCR reaction and displayed on the surface of the EBY100 strain of *S. cerevisiae* (Figure 1C). Following APEX labeling, yeast cells expressing the most active APEX2 mutants were selected and isolated using FACS. After four rounds of probe labeling and FACS selection, the R4‐Btn‐An library exhibited a noticeable increase in biotinylation signal compared to APEX2, whereas the R4‐BP5 library did not (Figure S2). The APEX2 library labeled with Btn‐An converged significantly toward the ^L242F^APEX2 variant (Figure S3). Conversely, for BP5 labeling, the most active mutants in the R4‐BP5 library remained predominantly APEX2 (Figure S3). Further validation on yeast surface demonstrated that ^L242F^APEX2 increased Btn‐An labeling activity by 0.5 orders of magnitude compared to APEX2 (Figure 1D). The L242 site is localized at the α‐helix of the APEX2 C‐terminus and is positioned toward the substrate binding pocket around the heme ligand (Figure 1E). The introduction of phenylalanine with an aromatic side chain into this pocket may strengthen enzyme‐substrate binding via π–π interactions. Sequence homology analysis revealed that the L242 site is highly conserved among different species, such as soybean, maize, and rice (Figure 1F). Moreover, this residue is also conserved in another class I peroxidase, yeast cytochrome c peroxidase (CcP), but not in class III peroxidase, HRP.

As we hypothesized that a mutant residue with an aromatic side chain could strengthen enzyme‐substrate binding via π–π interactions, we investigated whether other residues with aromatic side chains could also increase Btn‐An labeling intensity. Flow cytometry assay of yeast surface biotinylation revealed stronger labeling capacity when L242 was mutated to phenylalanine, tryptophan, or tyrosine, but not histidine (Figure S4). We speculated that phenylalanine largely stabilized the enzyme‐substrate interaction due to the presence of a benzene ring.

### 2.2 Characterization of ^L242F^APEX2 Mutant in Mammalian Cells

**FIGURE 2:**
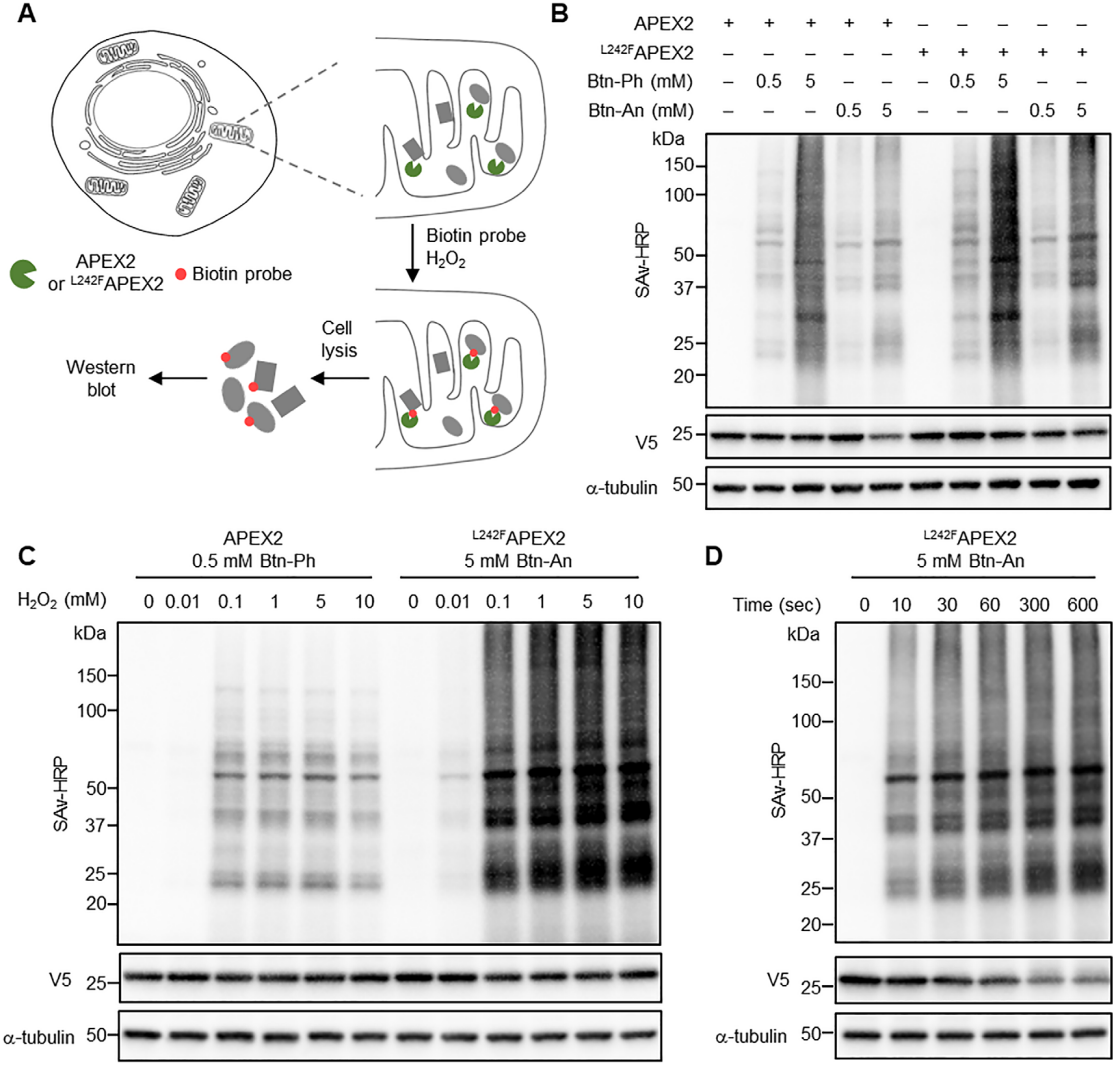
Test of ^L242F^APEX2 labeling toward proteins in mammalian cells. (A) Schematic representation of the workflow of protein labeling mediated by mito‐APEX2 or mito‐^L242F^APEX2 in mammalian cells. (B–D) Streptavidin blot analysis comparing protein biotinylation in HEK293T cells stably expressing APEX2 or ^L242F^APEX2 in the mitochondrial matrix, labeled with Btn‐Ph or Btn‐An at different probe concentrations (B), H_2_O_2_ concentrations (C), or for various durations (D).

Having demonstrated the improved activity of ^L242F^APEX2 on the yeast surface, we next conducted a direct comparison between ^L242F^APEX2 and APEX2 in mammalian cells. The peroxidase APEX2 or ^L242F^APEX2 was stably expressed in HEK293T cell mitochondrial matrix by fusing it with the mitochondrial targeting sequence of human cytochrome c oxidase subunit 4 (COX4), namely mito‐APEX2 and mito‐^L242F^APEX2, respectively (Figure 2A). Immunoblotting and RT‐qPCR analysis revealed that the expression levels of these two enzymes showed no statistically significant difference (Figure S5). Under labeling conditions of 500 µM Btn‐Ph, ^L242F^APEX2 already demonstrated higher labeling intensity than APEX2. The elevation was also significant for Btn‐An labeling, especially at a concentration of 5 mM. Compared with APEX2, ^L242F^APEX2 showed nearly a 2‐fold increase in labeling intensity (Figure 2B; Figure S6). Notably, ^L242F^APEX2 exhibited the strongest labeling signal in the presence of 5 mM Btn‐Ph. Furthermore, we noticed that ^L242F^APEX2 enhanced the labeling efficiency of both substrates, which is contradictory to the results on the yeast cell surface (Figure S4). This apparent discrepancy likely stems from differences in the local microenvironment, such as pH, ionic strength, and redox potential, between mammalian cells and the yeast cell surface.

Furthermore, we sought to optimize the hydrogen peroxide concentration for ^L242F^APEX2 labeling, as 1 mM H_2_O_2_ is typically used for proteomic APEX labeling. As demonstrated in Figure 2C, a similar labeling intensity was observed when 500 µM Btn‐Ph was paired with either 100 µM or 1 mM of H_2_O_2_. However, for 5 mM Btn‐An, 1 mM H_2_O_2_ outperforms 100 µM by 1.3‐fold. This comparison indicated that ^L242F^APEX2 labeling with Btn‐An exhibited stronger resistance to hydrogen peroxide than APEX2 labeling with the Btn‐Ph probe. Similar increases in hydrogen peroxide resistance were also reported in previous APEX‐directed evolution studies, revealing a resistance order of peroxidase from top to bottom: HRP, APEX2, APEX, and APX [6]. The increase in hydrogen peroxide resistivity can be explained by the lesser inactivation of the active Compound I intermediate due to the oxidation of H_2_O_2_, allowing more Compound I to participate in the oxidation of the aromatic substrates. Characterization of H_2_O_2_ concentration on yeast surface also aligned with the results observed in mammalian cells, indicating that ^L242F^APEX2 can achieve a higher biotinylation signal under 10 µM H_2_O_2_ than under 1 µM H_2_O_2_, whereas APEX2 cannot (Figure S7).

We also conducted experiments to determine the optimal labeling time for the ^L242F^APEX2 mutant. To our surprise, ^L242F^APEX2 labeling with Btn‐An achieved impressive biotinylation with as short as 10 s of H_2_O_2_ treatment (Figure 2D). We compared the efficacy of APEX2 and ^L242F^APEX2 in activating different concentrations of Btn‐An and Btn‐Ph probes within the 10‐s labeling window. Immunoblotting analysis indicated that ^L242F^APEX2 demonstrated an enhancement in the labeling efficiencies for both probes, a trend consistent with that observed under the 1‐min labeling condition (Figures S6 and S8).

Beyond efficiency, we assessed the cytotoxicity associated with radical‐based proximity labeling techniques. The cytotoxicity following exposure to high‐concentration Btn‐An, H_2_O_2_, and ^L242F^APEX2‐mediated labeling was assessed in wild‐type HEK293T and mito‐^L242F^APEX2 cells via cell proliferation assay. Btn‐An and H_2_O_2_ incubation alone had negligible or minimal impact on cell viability (Figure S9). In contrast, the complete ^L242F^APEX2‐mediated labeling procedure significantly reduced viability. However, since protein/RNA isolation immediately follows APEX labeling, the associated cytotoxicity was deemed acceptable for downstream applications.

Finally, we examined the subcellular distribution of ^L242F^APEX2. A recent study has identified the cytoplasmic‐biased localization of APEX2, which was attributed to a putative nuclear export signal (NES) proximal to the L242 residue [11]. Mutating this leucine to a less hydrophobic alanine (i.e., ^L242A^APEX2) eliminated the localization bias while preserving peroxidase activity. In our engineered variant, ^L242F^APEX2, the leucine was substituted with a more hydrophobic phenylalanine, a mutation hypothesized to enhance its intrinsic cytoplasmic targeting property. To test this, we generated HEK293T cell lines stably expressing EGFP‐tagged APEX2 or ^L242F^APEX2, and subsequently quantified the nucleocytoplasmic distribution of each variant based on EGFP fluorescence. Consistent with the previous report [11], APEX2‐EGFP exhibited a stronger cytoplasmic localization bias than EGFP alone. When the L242F mutation was introduced into APEX2, the resulting ^L242F^APEX2‐EGFP fusion exhibited a modest but statistically significant increase in cytoplasmic localization bias compared to APEX2‐EGFP (Figure S10). Treatment with leptomycin B, an inhibitor of XPO1‐dependent nuclear export [12], significantly increased the nuclear accumulation of ^L242F^APEX2‐EGFP, supporting the presence of a putative NES motif.

### 2.3 Higher Labeling Efficiency of ^L242F^APEX2 Mutant Toward RNA

After observing that ^L242F^APEX2 exhibits greater activity toward proteins than APEX2 in mammalian cells, we proceeded to investigate whether ^L242F^APEX2 could enhance RNA labeling efficiency. To accomplish this, we established three cell lines stably expressing ^L242F^APEX2 fusion proteins targeted to specific subcellular locations: (1) mitochondrial matrix; (2) the cytosolic face of the ER membrane, via fusion with Sec61β, a component of the ER translocon complex (^L242F^APEX2‐ERM); (3) cytoplasm, using a NES motif from HIV‐1 Rev protein (^L242F^APEX2‐NES). The localization of these fusion proteins was verified through immunostaining against corresponding organelle markers (Figure S11A). Additionally, for a direct comparison between APEX2 and ^L242F^APEX2, we employed previously‐established cell lines expressing mito‐APEX2, APEX2‐ERM, or APEX2‐NES, generated using identical targeting strategies [13].

**FIGURE 3:**
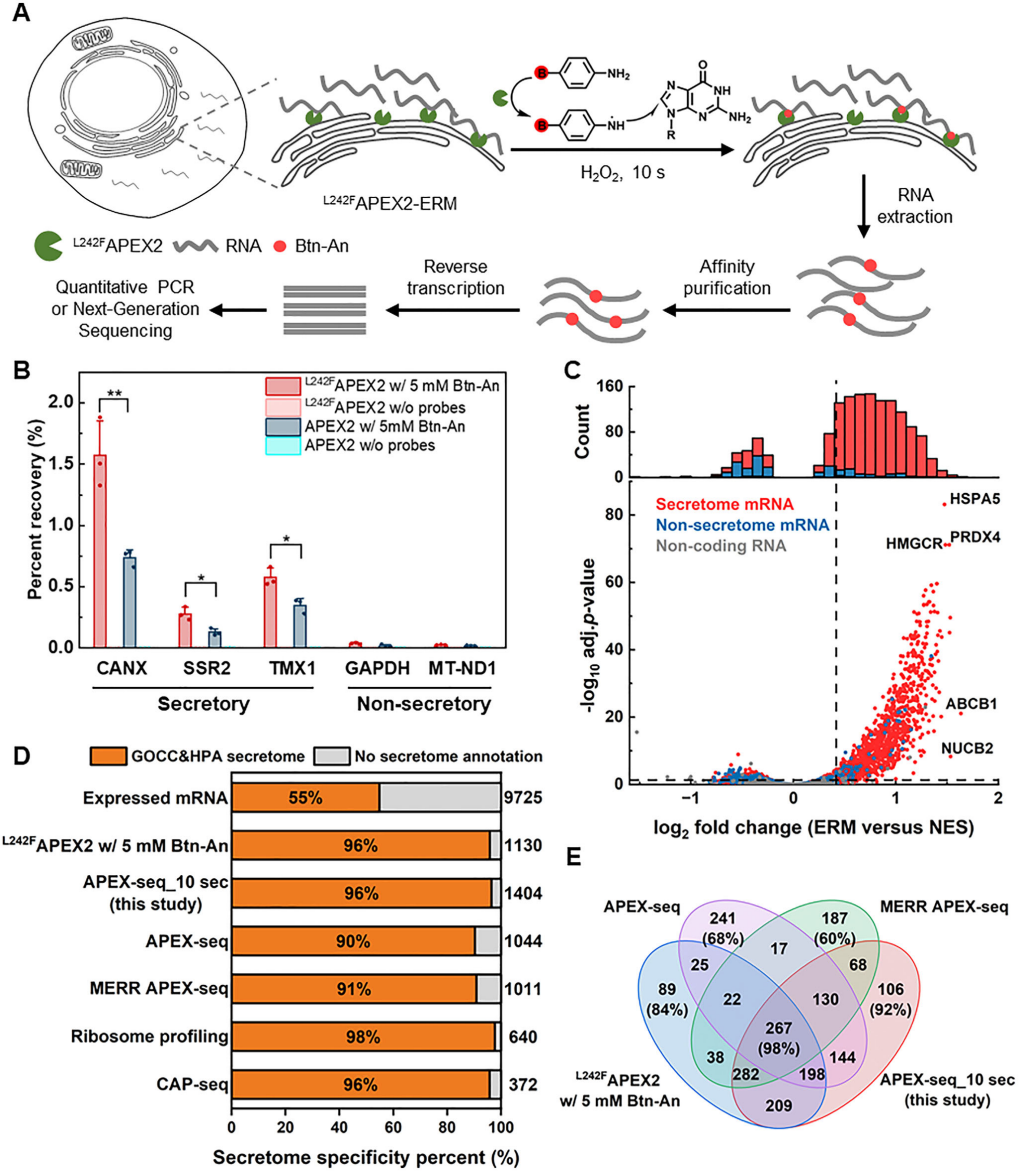
Profiling of ER membrane‐localized transcriptome with ^L242F^APEX2. (A) Schematic representation of the workflow of RNA labeling mediated by ^L242F^APEX2‐ERM in mammalian cells. (B) RT‐qPCR analysis of the recovery rates for RNAs labeled and enriched by APEX2 variants targeted to the ER membrane. Error bars represent SD. Statistical significance was calculated by a one‐sided *t‐*test. **: *p* < 0.01; *: *p* < 0.05. *n* = 3 biological replicates. (C) Volcano plot presenting ^L242F^APEX2‐mediated labeling of secretome mRNAs (red), non‐secretome mRNAs (blue), and non‐coding RNAs (grey). Horizontal dashed line indicates adj.*p*‐value = 0.05. Vertical dashed line indicates the cutoff of log_2_ fold change (ERM vs. NES) = 0.42. Upper panel, histograms showing the distribution of RNAs with adj.*p*‐value < 0.05. (D) Comparison of the spatial specificity of RNA labeling with various methods. Total mRNA denotes mRNAs with average counts more than 100 in the ^L242F^APEX2‐ERM cell line. (E) Venn diagram comparing the ER membrane transcriptomes profiled by peroxidase‐based proximity methods. The percentage in the parenthesis represents the ratio of secretome genes.

Next, we examined the spatial specificity of ^L242F^APEX2‐mediated labeling in the aforementioned three cell lines via immunofluorescence imaging. Each cell line was treated with 5 mM Btn‐An for 30 min, followed by a 10‐s biotinylation triggered by H_2_O_2_ (Figure 3A). Immunofluorescence imaging demonstrated co‐localization of Btn‐An labeling signals with APEX2 expression in all three cell lines (*r*_pearson, median_ = 0.7‐0.9), while negligible background was observed in the control samples where Btn‐An probe or H_2_O_2_ was omitted (Figure S11B–E).

After confirming the expression of ^L242F^APEX2 and its specificity in labeling, we characterized the labeling efficiency of ^L242F^APEX2 toward RNAs using RT‐qPCR as the readout. Biotinylated RNAs were extracted from labeled cells, enriched by streptavidin‐coated magnetic beads, and subjected to reverse transcription (Figure 3A). First, we observed that shortening the labeling time from 1 min to 10 s had a limited impact on the yield of biotinylated RNA while maintaining spatial specificity (Figure S12). Given its favorable kinetics and reduced cytotoxicity (Figure S9), we adopted the 10‐s labeling protocol for all subsequent applications.

Next, we compared the RNA labeling activity between ^L242F^APEX2 and APEX2 in the mitochondrial matrix and ER membrane. RT‐qPCR analysis of RNAs labeled by mito‐^L242F^APEX2 or mito‐APEX2 revealed that ^L242F^APEX2 recovered slightly more (∼1.6‐fold) MT‐mRNAs (*MT‐CO2*, *MT‐CYB*, and *MT‐ND1*) relative to APEX2 (Figure S13), consistent with ^L242F^APEX2‐mediated protein labeling. Notably, regardless of whether ^L242F^APEX2 or APEX2 was used, samples labeled with 5 mM Btn‐An yielded significantly more biotinylated RNAs than those labeled with 0.5 mM Btn‐Ph (Figure S13). Similarly, we observed that ^L242F^APEX2‐ERM showed moderate improvements (1.5∼2.2‐fold) in labeling mRNAs encoding membrane proteins (*CANX*, *SSR2*, and *TMX1*) compared with APEX2‐ERM (Figure 3B), despite the significantly lower expression level of ^L242F^APEX2‐ERM compared to APEX2‐ERM (Figure S14). Taken together, we concluded from the above data that ^L242F^APEX2 exhibited higher RNA labeling efficiency than APEX2.

Furthermore, we compared the RNA labeling efficiency of Btn‐An and Btn‐Ph probes at varying concentrations when catalyzed by ^L242F^APEX2. In both mitochondrial matrix and ER membrane compartments, RT‐qPCR analysis revealed that increasing the probe concentration from 0.5 to 5 mM significantly boosted labeling efficiency for both probes. However, the use of 5 mM Btn‐Ph, but not Btn‐An, was associated with a substantial rise in the off‐target transcript (*e.g. XIST*) recovery, suggesting a loss of spatial specificity (Figure S15).

### 2.4 Profiling Transcriptome Proximal to ER Membrane with ^L242F^APEX2

We proceeded to conduct a more comprehensive evaluation of the spatial specificity of ^L242F^APEX2‐mediated RNA labeling, focusing on the ER membrane as a model system. We performed three replicated RNA labeling experiments for both ^L242F^APEX2‐ERM and ^L242F^APEX2‐NES cell lines. The latter served as a non‐targeted control to eliminate background signals stemming from free radical diffusion. Pearson correlation analysis indicated highly reproducible enrichment of biotinylated RNAs from both cell lines (*r* > 0.99) (Figure S16A,B). Through receiver‐operating characteristic (ROC) analysis, we determined the threshold of log_2_ fold change (ERM vs. NES) to be 0.42 (Figure S16C; See Methods). This yielded a list of 1136 significantly‐enriched RNAs, including 1130 mRNAs (99.4%) (Figure 3C; Data S1). Gene Ontology Cellular Components (GOCC) analysis unveiled that proteins encoded by these mRNAs are predominantly associated with the secretory pathway (Figure S16D), which is consistent with the notion of localized translation at the ER membrane [14, 15].

The transcriptome associated with the ER membrane has been characterized using various methods including proximity‐specific ribosome profiling [16], APEX‐seq [17], CAP‐seq [18], and MERR APEX‐seq [13]. To assess the specificity of these methods, we compiled a list of genes related to the secretome based on annotations from GOCC [19, 20] and the Human Protein Atlas (HPA) [21] (see Methods). Our dataset included 1082 mRNAs (96%) from this list, demonstrating a level of specificity comparable to that of proximity‐specific ribosome profiling (98%) and CAP‐seq (96%) (Figure 3D). Notably, our method exhibited moderately superior specificity compared to APEX‐seq (90%) and MERR APEX‐seq (91%) (Figure 3D). Further analysis revealed that each peroxidase‐based method recovered a somewhat distinct pool of transcripts, likely influenced by the bias of different labeling mechanisms and APEX2 targeting strategies (Figure 3E).

A published APEX‐seq ERM dataset was generated via fusing APEX2 with the transmembrane segment of P450 oxidase 2C1 under 1‐min labeling conditions [17]. For a direct comparison of specificity and coverage between ^L242F^APEX2 and the classic APEX‐seq technique within the 10‐s labeling window, we performed labeling with 0.5 mM Btn‐Ph for 10 s in APEX2‐ERM and APEX2‐NES cell lines. Applying a similar analysis pipeline as above, ER membrane‐localized APEX2 significantly enriched 1412 RNAs, hereafter referred to as the APEX‐seq_10 sec dataset (Figure S17). This dataset exhibited slightly higher coverage and equally high secretome specificity (96%) compared to the ^L242F^APEX2 dataset (Figure 3D). An overlap analysis further revealed that 956 mRNAs (85%) identified by ^L242F^APEX2 were also captured in the APEX‐seq_10 sec dataset (Figure 3E).

Taken together, the combination of ^L242F^APEX2 with 5 mM Btn‐An probe offers higher efficiency in RNA labeling with comparable spatial specificity and coverage relative to APEX‐seq (Figure 3; Figures S16 and S17).

### 2.5 Profiling Midbody‐Proximal Transcriptome with ^L242F^APEX2

**FIGURE 4:**
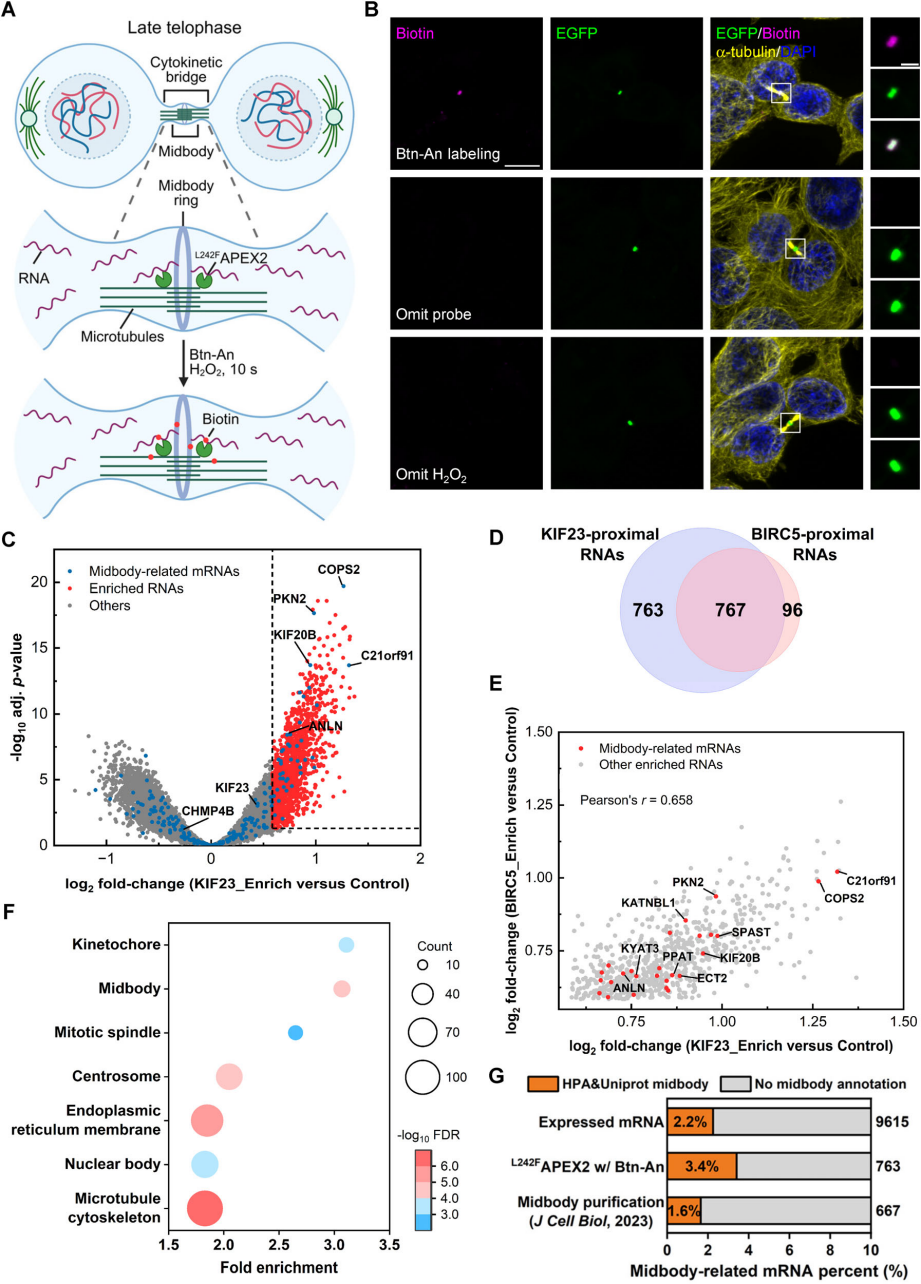
Mapping midbody‐proximal transcriptome with ^L242F^APEX2‐mediated RNA labeling. (A) Schematic of RNA proximity labeling mediated by midbody‐localized ^L242F^APEX2 during telophase. Created in BioRender. https://www.biorender.com/w26smjd (B) Representative immunofluorescence images showing the distribution of ^L242F^APEX2 (green), biotinylation (magenta), α‐tubulin (yellow), and DAPI‐stained DNA (blue) in synchronized KIF23‐^L242F^APEX2‐EGFP cells. Rows compare the complete labeling reaction (top) to controls omitting either the Btn‐An probe (middle) or H_2_O_2_ (bottom). The rightmost panels are magnified views of the boxed regions, displaying the biotinylation (top), EGFP (middle), and a merged image of both signals (bottom). Scale bars: 10 µm (overview); 2 µm (zoom‐in regions). (C) Volcano plot of RNAs enriched by KIF23‐^L242F^APEX2‐EGFP. Significantly enriched RNAs are shown in red, and midbody‐related mRNAs are highlighted in blue. Dashed lines indicate significance (horizontal, adj. *p*‐value = 0.05) and fold‐change (vertical, log_2_ fold‐change = 0.585) thresholds. (D) Overlap of RNAs significantly enriched by ^L242F^APEX2‐KIF23 and BIRC5‐^L242F^APEX2, respectively. (E) Correlation of enrichment levels for the overlapping RNAs identified in (D). Transcripts chosen for smFISH validation are labeled. (F) GOCC enrichment analysis of 763 midbody‐proximal mRNAs captured by ^L242F^APEX2. (G) Stacked bar plot showing the proportion of mRNAs encoding midbody‐localized proteins (annotated by HPA and Uniprot) within each dataset.

Having validated the spatial specificity of ^L242F^APEX2‐mediated labeling using the ER membrane model, we next applied this approach to a more challenging subcellular compartment, namely the midbody. The midbody is a protein‐rich structure assembled from the antiparallel central spindle microtubules during telophase (Figure 4A). It is essential for cytokinesis, serving as a scaffold that recruits and activates the abscission machinery [22, 23, 24]. Besides canonical abscission‐mediating proteins, proteomic analysis of purified midbodies revealed an enrichment of RNA binding proteins (RBPs) and RNA processing factors, implicating the presence of RNA at this site [25, 26, 27, 28]. Accordingly, recent studies have mapped midbody‐localized transcriptome via isolation of the midbody from synchronized cells, and identified ribonucleoprotein complexes and local translation [29, 30]. These observations prompted us to investigate the RNA composition at midbody using our enhanced proximity‐labeling method.

Given that the expression levels and subcellular localizations of most midbody protein components are tightly linked to the cell cycle, we employed CRISPR/Cas9‐mediated knock‐in to target ^L242F^APEX2 specifically to the midbody during telophase. This was achieved by fusing ^L242F^APEX2‐EGFP to the C‐terminus of endogenous midbody proteins. We selected two midbody protein markers for this purpose: (1) KIF23/MKLP1, a core structural component of the midbody ring [31], and (2) BIRC5/Survivin, which predominantly localizes to the plus end of the cytokinetic bridge and has been utilized in prior study for cytokinetic bridge‐proximal proteome profiling [32, 33] (Figure S18). A homozygous knock‐in clone for BIRC5 and a heterozygous knock‐in clone for KIF23 were isolated and expanded for downstream analysis, respectively (Figure S19). Immunofluorescence imaging confirmed the specific targeting of the ^L242F^APEX2 fusion proteins to the midbody during telophase (Figure S20).

Next, we evaluated the spatial specificity of ^L242F^APEX2 labeling in KIF23‐ ^L242F^APEX2 and BIRC5‐^L242F^APEX2 cell lines via immunofluorescence imaging. In late mitosis, Btn‐An labeling signal co‐localized precisely with ^L242F^APEX2 expression in both cell lines, without detectable nonspecific signals in other subcellular structures (Figure S21). As mitotic telophase spans a relatively short period within the cell cycle, we synchronized the two cell lines to late mitosis using a sequential block with mimosine and aphidicolin, followed by ^L242F^APEX2 labeling as outlined in Figure S22. Immunofluorescence analysis revealed that cell cycle synchronization did not affect the spatial specificity of Btn‐An labeling. In the negative control samples omitting the Btn‐An probe or H_2_O_2_, only the background signal from endogenous biotinylated proteins was observed. (Figure 4B; Figure S23).

To obtain transcriptomes specifically labeled by KIF23‐^L242F^APEX2 or BIRC5‐^L242F^APEX2 fusion proteins, we performed four biologically independent ^L242F^APEX2 labeling experiments in each synchronized knock‐in cell line. Enrichment of biotinylated RNAs was highly reproducible across replicates, as demonstrated by high Pearson's correlation coefficients (> 0.97) (Figure S24). To identify transcripts proximal to the midbody, we performed DESeq2 [34] analysis comparing RNAs enriched from midbody‐localized ^L242F^APEX2 samples (Enrich) vs. those from unlabeled samples omitting the H_2_O_2_ trigger (Control). Using a threshold of fold‐change > 1.5 and FDR‐adjusted *p*‐value < 0.05, we identified 1530 and 863 RNAs significantly enriched by the two ^L242F^APEX2 fusion proteins, respectively (Figure 4C; Figure S25A). The intersection of these two datasets yielded 767 RNAs, which were defined as the midbody‐proximal transcriptome in this study (Figure 4D). The enrichment levels and normalized abundances of these 767 RNAs were highly correlated between the two DESeq2 comparisons, indicating consistent enrichment by midbody‐localized ^L242F^APEX2 (Figure 4E; Figure S25B). GOCC and GOBP analyses revealed that proteins encoded by the enriched RNAs were primarily localized to midbody‐associated structures and involved in the regulation of mitosis (Figure 4F; Figure S25C).

Among the 767 midbody‐proximal RNAs, 26 mRNAs encode proteins with annotated midbody localization (hereafter designated as midbody‐related mRNAs, see Methods). The proportion of midbody‐related mRNAs (3.4%) was higher than that in mRNAs expressed in both knock‐in cell lines (2.2%) or in RNAs captured by midbody purification (1.6%) [30] (Figure 4G). Although proximity labeling and midbody purification captured relatively few common RNAs, we observed that the transcriptome captured by biochemical fractionation was contaminated with mitochondrial rRNAs (i.e., *MT‐RNR1* and *MT‐RNR2*) (Figure S25D). Two previously‐reported midbody‐localized mRNAs, *KIF23* ^29^ and *CHMP4B^30^*, were not detected in our dataset (Figure 4C; Figure S25A). Their absence at the midbody during telophase was confirmed by single‐molecule RNA FISH (smFISH), suggesting cell‐type‐specific RNA localization (Figure S26A).

### 2.6 Translation‐Dependent Targeting of *ANLN* mRNA to the Midbody

**FIGURE 5:**
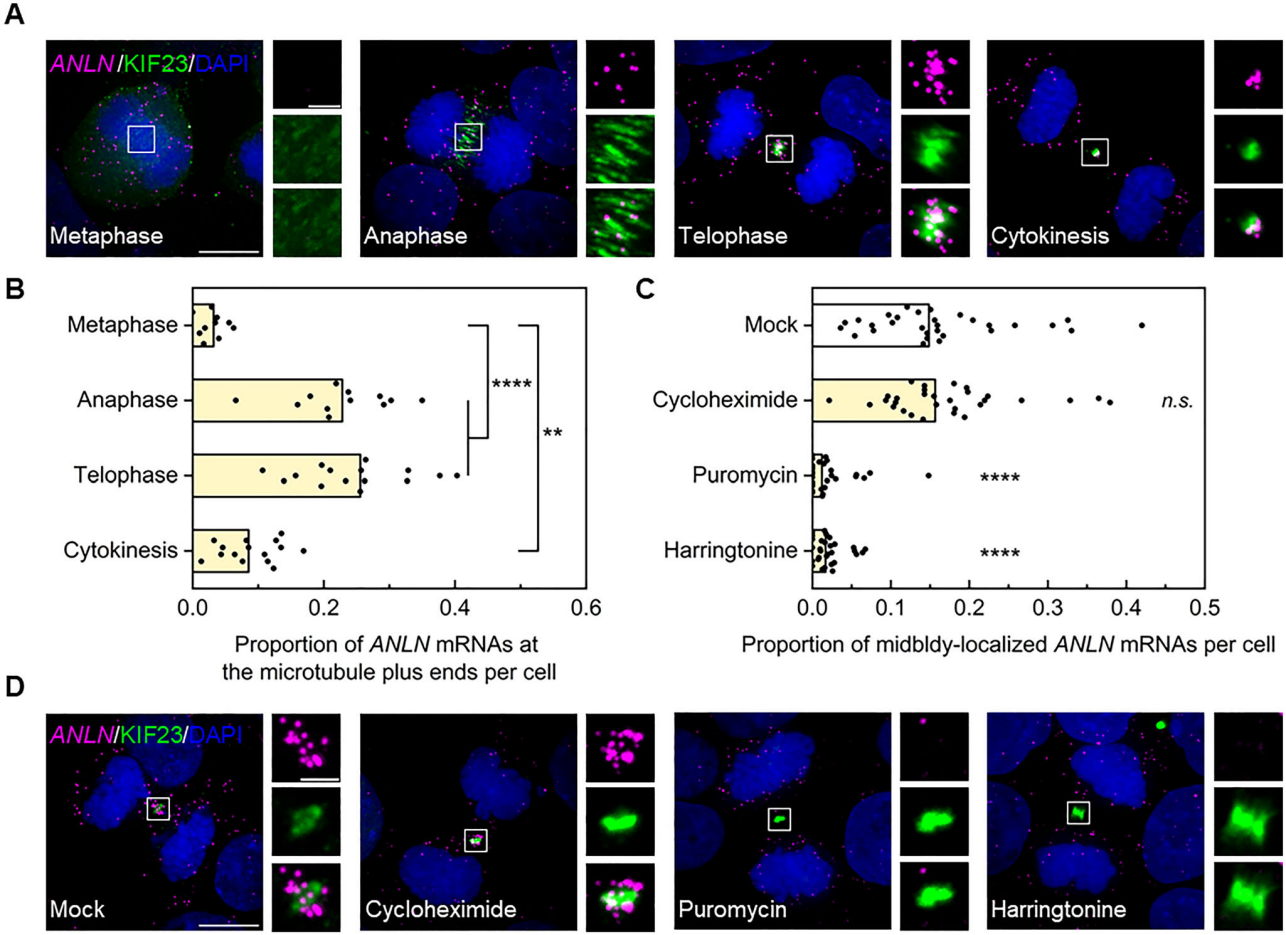
Imaging analysis of *ANLN* mRNA localization during the cell cycle and upon translation inhibition. (A,D) Representative smFISH images of endogenous *ANLN* mRNA in HEK293T cells at the indicated cell cycle phases (A) or following treatment with distinct translation inhibitors (D). Magenta: endogenous *ANLN* mRNA; green: KIF23 immunofluorescence to visualize the midbody; blue: DNA stained with DAPI. Magnified views of the boxed region are shown in the right, displaying RNA (top), KIF23 (middle), and a merged image of both (bottom). Scale bars: 10 µm (overview); 2 µm (zoom‐in regions). (B,C) Bar plots show the median proportion of midbody‐localized *ANLN* mRNA per cell. For (B), data were collected from 10, 12, 15, and 15 cells across three biological replicates. For (C), data were collected from 30 cells in two biological replicates. Cells treated with DMSO served as the reference. Statistical significance was determined by a two‐sided Mann–Whitney test. *n.s*.: not significant. **: *p* < 0.01, ****: *p* < 0.0001.

To validate our midbody‐proximal RNA dataset, we selected 10 enriched midbody‐related mRNAs, including those with high enrichment levels (*C21orf91*, *COPS2*, *PKN2*) or high normalized abundances (*ANLN*, *ECT2*) (Figure 4E; Figure S25B), for further smFISH imaging analysis. Unexpectedly, most of these transcripts did not show detectable midbody localization during telophase (Figure S26B,C). However, *ANLN* mRNA was confirmed as a novel midbody‐localized transcript via smFISH (Figure 5A,B). We therefore focused on *ANLN* mRNA for further analysis.

Anillin protein, encoded by *ANLN* mRNA, is a conserved scaffold protein that interacts with cytoskeletal components and their regulators, and plays a key role in the cytokinesis as part of the contractile ring [35, 36, 37]. smFISH imaging not only verified the midbody enrichment of *ANLN* mRNA but also revealed its highly dynamic subcellular localization during mitosis. During metaphase, *ANLN* mRNA was distributed throughout the whole cell. When cells enter the anaphase, the accumulation of mRNA at the spindle midzone became pronounced. As cells progressed into telophase, *ANLN* mRNA became concentrated at the midbody, marked by KIF23 protein, and remained enriched until cytokinesis completion (Figure 5A,B). Given that the ANLN protein also localizes to the midbody, we investigated the spatial relationship between the mRNA and its protein product during mitosis. Imaging uncovered partial co‐localization of *ANLN* mRNA and ANLN protein upon the formation of the cleavage furrow. During cytokinesis, both were enriched at the midbody, implicating the possibility of local translation (Figure S27).

We next asked whether the midbody localization of *ANLN* mRNA during telophase is conserved across cell types. To this end, smFISH imaging was performed in four human cancer cell lines: HeLa, U‐2 OS, SH‐SY5Y, and MCF‐7. In contrast to HEK293T cells, no apparent midbody accumulation of *ANLN* mRNA was observed in any of these cell lines during late telophase (Figure S28). Nevertheless, transient mRNA localization to the spindle midzone in early telophase was detected in MCF‐7 cells, mirroring the observation in HEK293T cells. These results suggest that the subcellular localization of *ANLN* mRNA is cell‐type‐specific.

To explore the mechanism underlying midbody localization of *ANLN* mRNA, we considered the emerging role of co‐translational targeting in directing RNAs to specific subcellular compartments such as ER membrane [14], outer mitochondrial membrane [38], and centrosome [39, 40]. Therefore, we tested whether *ANLN* mRNA localization depends on translation. HEK293T cells were treated with three distinct translation inhibitors: cycloheximide, puromycin, and harringtonine. Though all three drugs inhibit translation elongation or initiation, they differ in their effects on the polysome integrity: cycloheximide stabilizes polysomes, puromycin disrupts polysomes and releases mRNAs [41, 42], and harringtonine traps the initiating ribosome at the start codon and prevents polysome assembly [43].

smFISH imaging following drug perturbation revealed that puromycin and harringtonine significantly weakened midbody targeting of *ANLN* mRNA, whereas cycloheximide had little effect (Figure 5C,D). Since nascent peptides remain associated with ribosomes after cycloheximide but not puromycin treatment, we speculated that midbody localization of *ANLN* mRNA requires the nascent polypeptides. Moreover, the preserved localization under cycloheximide treatment suggests once the polysomes are assembled and maintained, ongoing translational elongation is not required to sustain *ANLN* mRNA at the midbody.

### 2.7 Requirement of N‐Terminal Nascent Polypeptide for *ANLN* mRNA Localization

**FIGURE 6:**
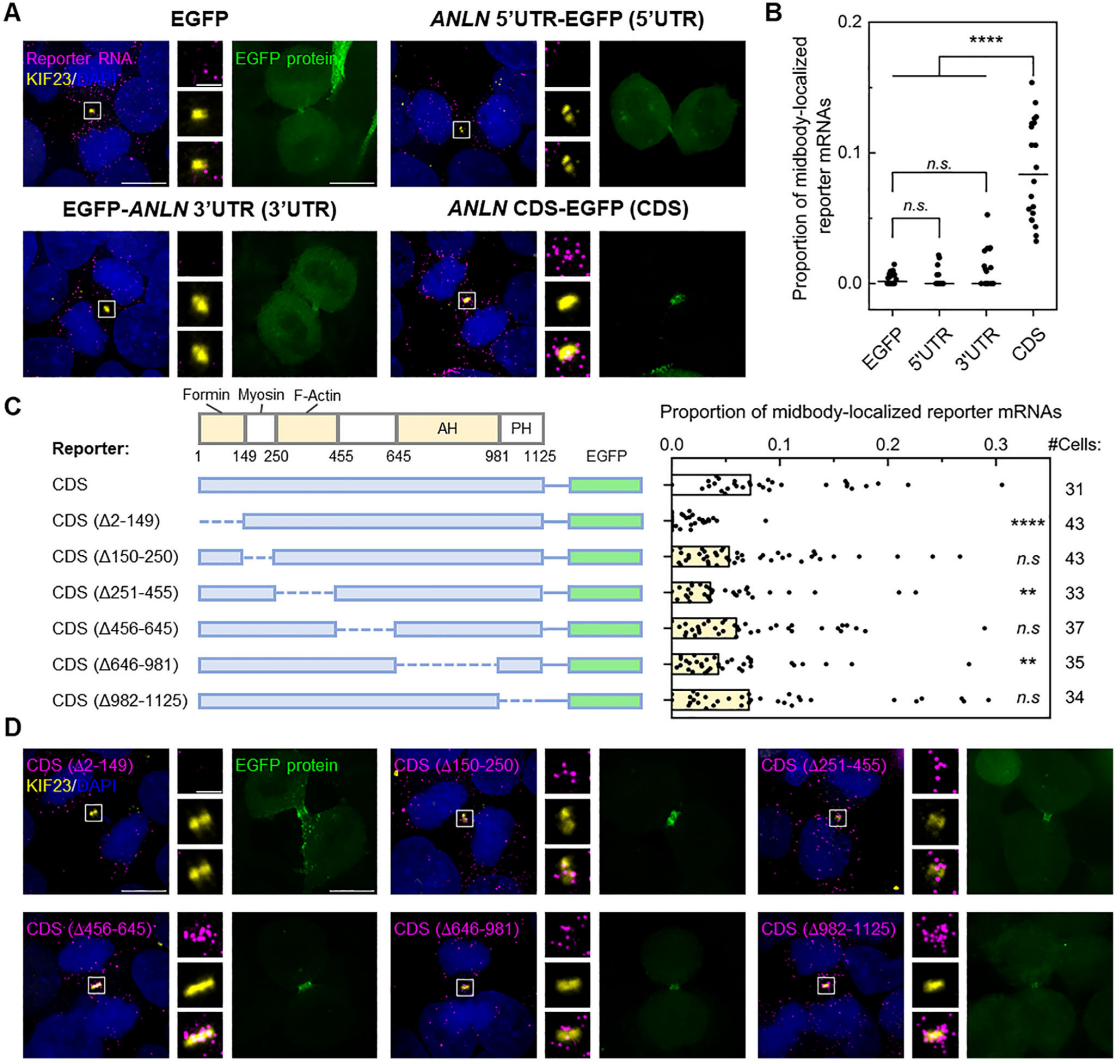
Investigation of RNA localization elements within *ANLN* mRNA using EGFP reporter fusions. (A and D) Representative images of mitotic HEK293T cells transiently expressing the indicated EGFP reporter constructs. Magenta: EGFP reporter mRNA; yellow: KIF23 immunofluorescence; blue: DNA stained with DAPI; green: EGFP fusion proteins. Magnified images of the boxed region display reporter mRNA (top), KIF23 (middle), and a merged image of both (bottom). Scale bars: 10 µm (overview); 2 µm (zoom‐in regions). (B) Grouped scatter plot shows the proportion of midbody‐localized reporter mRNAs for constructs in (A). Data were collected from 20 cells in two biological replicates. (C) Left: schematic of *ANLN* CDS deletions for generating EGFP reporter constructs. The dashed lines indicate deleted regions. Right: Bar plot shows the median proportion of midbody‐localized reporter mRNAs for constructs in (D). Cells expressing *ANLN* CDS‐EGFP served as the reference. The number of cells analyzed per construct was indicated on the graph (from two biological replicates). Statistical analysis for (B,C) was determined by a two‐sided Mann–Whitney test. *n.s*.: not significant. **: *p* < 0.01, ****: *p* < 0.0001.

To further elucidate how *ANLN* mRNA was transported to the midbody, we constructed a series of EGFP reporter mRNAs to identify the RNA region required for midbody localization. We first tested whether the untranslated regions (UTRs) of *ANLN* mRNA were capable of targeting EGFP reporter mRNAs to the midbody. The EGFP open reading frame (ORF) was fused to either the 5’UTR (ANLN 5’UTR‐EGFP) or the 3’UTR (EGFP‐ANLN 3’UTR) of *ANLN* mRNA. When these reporters were transiently expressed in HEK293T cells and detected using EGFP‐specific smFISH probes, neither reporter mRNA exhibited enrichment at the midbody during telophase (Figure 6A,B). In contrast, when fused to the *ANLN* coding sequence (ANLN CDS‐EGFP), the EGFP reporter showed pronounced midbody localization. Moreover, similar to endogenous ANLN protein (Figure S27), the resulting ANLN‐EGFP fusion proteins also co‐localized with the midbody (Figure 6A,B). As a negative control, mRNAs encoding EGFP alone were diffusely distributed throughout the cytoplasm (Figure 6A,B). Taken together, these results demonstrated that the CDS, but not the UTRs, of *ANLN* mRNA is indispensable for its midbody localization, which is consistent with a translation‐dependent localization mechanism revealed by drug perturbation. Interestingly, a previous study identified the 3’UTR of *CHMP4B* mRNA as the primary determinant for its midbody targeting in HeLa cells [30]. The distinct mechanisms governing *ANLN* and *CHMP4B* mRNA localization highlight the context‐dependent nature of midbody targeting.

Building on the finding that *ANLN* CDS is required for midbody localization, we next aimed to map the specific motifs/domains within the CDS responsible for mRNA targeting. Anillin is a well‐characterized modular scaffold protein: its N‐terminus contains regions for binding Formin (1‐149 aa), Myosin (150‐250 aa), and F‐actin (251‐455 aa), while its C‐terminus comprises an Anillin homology (AH) domain (646‐981 aa) and a Pleckstrin homology (PH) domain (982‐1125 aa) [36, 37]. Based on this domain architecture, we designed a panel of EGFP reporter constructs harboring deletions of individual CDS segments from the full‐length *ANLN* CDS (Figure 6C) to identify the peptide segments essential for mRNA localization. Removal of the Formin‐binding region (Δ2‐149 aa) abolished the enrichment of reporter mRNA at the midbody, whereas deletion of other segments did not (Figure 6C,D). Notably, the corresponding truncated EGFP fusion proteins retained their midbody localization, indicating that mRNA and protein targeting rely on distinct mechanisms.

Having demonstrated the necessity of the N‐terminal Formin‐binding region for *ANLN* mRNA localization, we next examined its sufficiency. To this end, we fused the CDS of the Formin‐binding region to the N‐terminus of EGFP ORF. Though the EGFP fusion protein products co‐localized with the midbody, the mRNA itself did not exhibit midbody enrichment (Figure S29). As we previously discovered that the centrosomal‐targeting efficiency of *DLGAP5* mRNA is tightly linked with its CDS length [40], we extended the reporter by appending additional ORFs (EGFP and HaloTag). The elongated reporter mRNA still failed to localize to the midbody (Figure S29). Together, these results suggest that while the Formin‐binding region of *ANLN* CDS is necessary for mRNA targeting, it is not sufficient on its own. Full targeting of mRNA to the midbody likely requires additional *cis*‐elements elsewhere in the CDS and/or higher‐order structural features of the CDS.

## 3 Discussions

Previous directed evolution efforts primarily focused on optimizing APEX activity using Btn‐Ph as the substrate [6]. Although novel substrates such as Btn‐An have been developed, engineering of APEX variants with enhanced activity toward these substrates remains unexplored. In this study, we performed yeast surface display‐based directed evolution using Btn‐An as the primary substrate and identified the L242F mutation in APEX2. This variant exhibits an approximately two‐fold increase in labeling efficiency and improved tolerance to H_2_O_2_. This enhancement likely arises from π–π interactions between the aniline probe and the newly introduced aromatic side chain at the 242 site. This hypothesis is further supported by the similar activity boost observed when leucine was substituted with other aromatic residues, such as tryptophan or tyrosine. The precise structural mechanisms by which the L242F mutation enhances activity, and whether it differentially influences the binding or activation of Btn‐An vs. Btn‐Ph, remains an open question that would benefit from future structural studies or molecular dynamics simulations.

In this study, we have also demonstrated that applying a high concentration (5 mM) of Btn‐An substantially enhances the labeling efficiency toward RNAs and proteins while preserving spatial specificity. The combination of our engineered ^L242F^APEX2 with 5 mM Btn‐An thus provides superior sensitivity, enabling effective capture even from limited experimental material. We first validated the high spatial specificity of ^L242F^APEX2‐mediated labeling at the ER membrane. Our method captured a total of 1136 transcripts with 96% specificity of genes encoding secretory pathway components, achieving comparable coverage and specificity to established proximity labeling methods [13, 16, 17, 18]. We next applied ^L242F^APEX2 to profile the transcriptome proximal to the midbody, a transient and dense structure assembled during late mitosis. By fusing ^L242F^APEX2 to two endogenous midbody‐localized proteins, we identified 767 midbody‐proximal transcripts from cell‐cycle synchronized HEK293T cells. Our dataset enriched transcripts encoding proteins that localize to the midbody and regulate mitosis, implicating the model of local translation at this cytokinetic structure as previous studies suggested [29, 30].

From the midbody‐localized transcripts, we confirmed the midbody localization of *ANLN* mRNA by smFISH imaging in HEK293T cells. Mechanically, our study revealed that its localization depends on a co‐translational targeting process and requires the nascent polypeptide translated from the *ANLN* CDS, rather than 3’UTR or specific RBPs reported for other midbody‐localized mRNAs [29, 30]. This mechanism resembles that reported for transcripts targeting to another mitosis‐related structure (e.g., the centrosome [39, 40]). Both the midbody and centrosome undergo rapid compositional and morphological remodeling during mitosis, suggesting that mRNA localization might provide an efficient approach for transporting large scaffold proteins to these sites. Through systematic CDS deletions, we further identified the N‐terminal Formin‐binding region as a necessary module for *ANLN* mRNA localization. Nevertheless, this region alone was insufficient to target a reporter mRNA to the midbody, implicating the involvement of additional regulatory elements. The *trans*‐acting factors that recognize this nascent peptide signal remain to be identified.

Finally, the boosted activity of ^L242F^APEX2/Btn‐An system allows reliable labeling within an ultrafast 10‐s window. This rapid labeling capability establishes ^L242F^APEX2 as a powerful tool for high temporal resolution studies, opening avenues to investigate fast biological processes. Although the present work focuses primarily on subcellular transcriptome profiling, the kinetic advantage of 10‐s labeling should prove valuable in proteomic applications, such as capturing rapid interactome rearrangements in signaling pathways. Additionally, the shortened labeling duration might lead to reduced cytotoxicity compared to longer H_2_O_2_ exposures, rendering the protocol suitable for use in H_2_O_2_‐sensitive systems.

## 4 Methods

### 4.1 Reagents and Resources

A full description of reagents and resources utilized in this study is listed in Table S1.

### 4.2 Yeast Strain and Media

*S. cerevisiae* EBY100 strain (MATα ura3‐52 trp1 leu2∆1 his3∆200 pep4::HIS3 prb1∆1.6R can1 GAL, pIU211:URA3) was a gift from Prof. Tao Liu at Peking University. Yeast transformation was performed with the EZ frozen transformation kit (Zymo Research). Yeast cells were grown in the synthetic drop‐out medium without tryptophan (SD media, containing 20 g/L glucose, 6.7 g/L yeast nitrogen base, and 1.76 g/L yeast synthetic drop‐out medium supplements without tryptophan and uracil) to mid‐log phase at 30°C on a rotary shaker. Protein expression was induced by culturing in yeast extract peptone galactose media (YPG media, containing 20 g/L galactose, 20 g/L peptone, and 10 g/L yeast extract).

### 4.3 Construction of APEX2 Random Mutant Library

The APEX2 random mutant library was created using an error‐prone PCR reaction. In this process, a 20 ng APEX2 fragment underwent 10 rounds of PCR amplification. The reaction included 0.4 µM of both forward and reverse primers, 0.2 mM dNTP, 2 mM MgCl_2_, 2 µM 8‐oxo‐dGTP, 2 µM dPTP, and 1 U of Taq DNA polymerase. The resulting PCR product was then purified using the DNA Clean and Concentration Kit (Zymo Research). Subsequently, the homologous arms of the APEX2 random mutant fragment were incorporated through another conventional PCR reaction using Taq DNA polymerase. The pCTcon2 vector was prepared by cleaving it with the restriction endonucleases NheI‐HF and BamHI‐HF. Then, 5 µg of the APEX2 random mutant fragment and 1 µg of the vector were mixed, purified with ethanol, and reconstituted in 10 µL of H_2_O.

Electrocompetent *S. cerevisiae* EBY100 cells were generated using the lithium acetate‐sorbitol method. Wild‐type EBY100 cells were cultured in YPD media until reaching an OD_600_ between 2.5 and 3. Cells were then resuspended in 100 mM lithium acetate and 10 mM dithiothreitol, and incubated at 30°C for 12 min. Following centrifugation at 3000 rpm for 3 min, the cell pellets were washed and resuspended in 1 M cold sorbitol solution.

The DNA library mix was electroporated into the prepared electrocompetent *S. cerevisiae* EBY100 cells using an electroporator (Bio‐Rad). The electroporated cells were transferred into a 12‐mL tube and allowed to recover at 30°C for 1 h without agitation. Subsequently, the recovered cells were cultured in 100 mL of SD media for further amplification.

### 4.4 APEX Labeling on Yeast Surface and FACS Analysis

All transformants were cultured until reaching mid‐log phase in SD cell culture, after which they were inoculated into YPG culture. At mid‐log phase, 0.33 mL of cells were harvested, washed twice with PBSB buffer (PBS buffer containing 0.1% BSA), and resuspended in 900 µL of PBSB buffer. The yeast suspension was then treated with 100 µL of a 10× mixture containing 1 mM probe and 10 µM H_2_O_2_ in PBSB for 1 min. After labeling, 1 mL 2× quencher buffer was added to the cells. Btn‐Ph, Btn‐An, and Btn‐Nap probes utilized in this study were synthesized according to the previous protocol [8]. The BP5 probe was a generous gift from Prof. Ruijun Tian, Southern University of Science and Technology.

The cells were subsequently washed twice with PBSB and resuspended in 50 µL of mouse anti‐V5 monoclonal antibody (diluted 1:200 in PBSB buffer) and incubated at room temperature for 1 h. Following incubation, the cells were washed with PBSB buffer twice and incubated with 50 µL secondary antibody solution (Alexa Fluor 647‐conjugated Streptavidin, diluted 1:200, and Alexa Fluor 488‐conjugated anti‐mouse goat IgG (H+L), diluted 1:500 in PBSB buffer). After antibody incubation, the cells were washed twice with PBSB buffer and resuspended in 200 µL PBSB. The fluorescent signals were then detected using Cytoflex Flow Cytometry, and the data were analyzed using FlowJo (v10.6.2).

### 4.5 FACS Selection

For the first‐round of selection of the R0 library, 20 mL of cells in mid‐log phase grown in YPG media were harvested for further APEX labeling. For the other rounds of selection, only 2 mL of cells in YPG media containing 1 mM succinyl acetone were needed. The APEX labeling and antibody staining protocol was identical to that for FACS analysis.

During FACS selection, biotinylated yeast cells were sorted using either a BD Aria III or BD Aria SORP sorter coupled with a laser for FITC and PE. For each round of selection, approximately 1% to 3% of highly active cells were sorted for further amplification.

### 4.6 Mammalian Cell Culture

HEK293T, HeLa, SH‐SY5Y, and MCF‐7 cell lines were cultured in Dulbecco's Modified Eagle's Medium (DMEM) supplemented with 10% fetal bovine serum (FBS). U‐2 OS cell line was cultured in McCoy's 5A Medium supplemented with 10% FBS. The HEK293T, HeLa, SH‐SY5Y, and U‐2 OS cell lines were obtained from the National Infrastructure of Cell Line Resource, Beijing, China. The MCF‐7 cell line was a generous gift from Prof. Mo Li, Peking University Third Hospital. All cell lines were maintained at 37°C in a humidified atmosphere containing 5% CO_2_.

### 4.7 Genetic Plasmid Construction

The plasmids pLX304‐mito‐V5‐^L242F^APEX2, pLX304‐V5‐^L242F^APEX2‐NES‐EGFP, and pLX304‐V5‐^L242F^APEX2‐Sec61b were generated by introducing a single‐point mutation into their corresponding APEX2 plasmids.

To generate knock‐in cell lines, the ^L242F^APEX2‐EGFP fragment was inserted into the endogenous *KIF23* or *BIRC5* locus using CRISPR/Cas9‐mediated homology‐directed repair. Guide RNAs (gRNAs) targeting sequences near the stop codon of each gene were designed with CRISPOR [44], and cloned into BsmBI‐digested lentiCRISPRv2 backbone (Addgene #98290, kindly provided by Prof. Mo Li) for co‐expression with Cas9. For each gene, two gRNAs were designed to target the sense and antisense strands, respectively. Donor plasmids were constructed by assembling the ^L242F^APEX2‐EGFP fragment, flanked by approximately 1‐kb homology arms derived from the *KIF23* or *BIRC5* genomic locus, into a pUC19 backbone. The gRNA sequences are provided in Table S4.

For the construction of EGFP reporters, *ANLN* CDS was amplified from a cDNA library synthesized by reverse transcription of total RNA from HEK293T cells using ProtoScript II First Strand cDNA Synthesis Kit. The *ANLN* 5’UTR and 3’UTR were amplified from genomic DNA of HEK293T cells. These fragments, including full‐length CDS, UTRs, or truncated CDS, were then assembled into the backbone of pUbC‐stdMCP‐stdGFP (Addgene #98916, kindly provided by Prof. Wulan Deng, Peking University). Details of the EGFP reporter plasmids used in this study are provided in Table S5.

All PCRs were performed with Phanta Max Super‐Fidelity DNA Polymerase, and the fragments were assembled via Gibson assembly using the Lightening Cloning Kit. The DNA sequences of these plasmids were confirmed by Sanger sequencing at Genewiz (Tianjin, China).

### 4.8 Generation of HEK293T Cell Lines Stably Expressing ^L242F^APEX2 Fusion Proteins

HEK293T cells stably expressing ER‐HRP, mito‐APEX2, APEX2‐NES, and APEX2‐ERM fusion proteins have been reported in previous studies [13, 45]. To establish cells stably expressing mito‐^L242F^APEX2, ^L242F^APEX2‐ERM, ^L242F^APEX2‐NES, APEX2‐EGFP, ^L242F^APEX2‐EGFP, ^L242F^APEX2‐EGFP‐3×NLS or EGFP, HEK293T cells seeded in a 6‐well plate at approximately 80% confluency were transfected with the corresponding lentiviral vector (1 µg) along with two packaging plasmids, pVSVG (0.7 µg) and dR8.91 (1 µg), using Lipofectamine 3000 transfection reagent (5.4 µL) in a total volume of 200 µL Opti‐MEM I reduced serum medium. Following 6 h of transfection, the medium was replaced with fresh complete medium.

After 48 h of transfection, the lentivirus‐containing medium was collected and filtered through a 0.45 µm syringe filter. Subsequently, 1 mL of lentivirus‐containing medium was added to wild‐type HEK293T cell cultures in a 6‐well plate. Following a two‐day lentiviral infection period, the medium was switched to complete medium supplemented with 1 µg/mL puromycin. Infected cells were maintained in complete medium containing 1 µg/mL puromycin for approximately 7 days. Immunofluorescence imaging was then employed to confirm the expression of ^L242F^APEX2 fusion proteins.

### 4.9 Generation and Genotyping of HEK293T Knock‐in Cell Lines

To establish the knock‐in cell lines, HEK293T cells were cultured in a 6‐cm dish until approximately 70% confluent and then transfected with the two gRNA vectors (1 µg for each) and donor plasmids (2 µg), using Lipofectamine 3000 transfection reagent (8 µL). After 6 h, the medium was replaced with fresh complete medium. At 7–9 days post‐transfection, single EGFP‐positive clones were isolated by FACS and expanded in 96‐well plates. After 12–14 days, the clones were transferred to 24‐well plates for further expansion and downstream characterization.

For genotyping, genomic DNA was extracted from each surviving clone. Target regions were amplified by PCR using the primers listed in Table S4, and the products were analyzed by agarose gel electrophoresis. Amplified fragments of the expected size were gel‐purified, cloned into a pUC19 backbone, and verified by Sanger sequencing.

### 4.10 Cell Cycle Synchronization

To synchronize HEK293T knock‐in cells to early S‐phase, a sequential block with mimosine and aphidicolin was applied. Briefly, when reaching approximately 30% confluency, cells were treated with 0.2 mM mimosine for 16 h, released for 10 h, and then blocked again with 2 µg/mL aphidicolin for 15 h. The aphidicolin block was removed by washing the cells three times with pre‐warmed DMEM. Subsequently, cells were released for 10.5 h to allow progression into late mitosis, followed by a 30‐min incubation with 5 mM Btn‐An dissolved in complete medium to enable ^L242F^APEX2‐mediated labeling.

For flow cytometry analysis of cell‐cycle phases, cells were harvested at specified time points, followed by fixation and permeabilization with ice‐cold 70% ethanol. Then, cells were incubated at 37°C for 30 min in PBS containing 0.1 mg/mL propidium iodide (PI), 0.1 mg/mL RNase A, and 0.1% Triton X‐100. The PI‐stained samples were analyzed on a CytoFLEX LX Flow Cytometer (Beckman), and the cell‐cycle distributions were estimated by FlowJo software.

### 4.11 ^L242F^APEX2‐Mediated Labeling in Living Cells

HEK293T cells stably expressing APEX2 or ^L242F^APEX2 fusion proteins at approximately 90% confluency were incubated with Btn‐An or Btn‐Ph probes (0.5 or 5 mM as indicated) for 30 min at 37°C in an environment containing 5% CO_2_. Biotinylation was initiated by adding 1 mM H_2_O_2_ for 1 min or 10 s, followed by treatment with a quencher solution containing 10 mM sodium ascorbate, 10 mM sodium azide, and 5 mM Trolox. After three rounds of quenching, the cells were either fixed for imaging, or lysed for immunoblotting or RNA extraction.

### 4.12 Immunofluorescence Microscopy

Following ^L242F^APEX2‐mediated labeling, cells were fixed and permeabilized with pre‐chilled methanol (−20°C) for 10 min at room temperature. After three washes, cells were incubated with blocking buffer (0.5% Tween‐20 and 3% BSA in PBS) at room temperature for 1 h. Subsequently, cells were incubated with primary antibodies (as specified below) diluted in blocking buffer at 4°C overnight. After washing three times with PBST (0.5% Tween‐20 in PBS), cells were further incubated with secondary antibodies (as specified below) diluted in PBS for 1 h at room temperature. Following three additional washes with PBST, cells were stained with DAPI diluted in PBS for 15 min, washed twice with PBS, and then stored in PBS at 4°C before imaging.

For general immunostaining, cells were fixed with 4% formaldehyde in PBS at room temperature for 20 min and washed three times with PBS. Subsequently, cells were permeabilized with 0.2% Triton X‐100 in PBS for 15 min. After three washes with PBS, cells were blocked, incubated sequentially with primary and secondary antibodies, and counterstained with DAPI as described previously.

The primary antibodies used in this study were as follows: mouse V5‐tag monoclonal antibody (3C8) (1:800, Biodragon, B1005), rabbit anti‐HSP60 antibody (1:250, abcam, ab46798), rabbit anti‐Calnexin antibody (1:250, abcam, ab22595), rat anti‐α‐tubulin antibody (YL1/2) (1:500, Invitrogen, MA180017), rabbit anti‐MKLP1/KIF23 antibody (1:500, abcam, ab174304), rabbit anti‐Anillin antibody (1:2000, CST, 48298), and anti‐Survivin/BIRC5 antibody (1:500, CST, 2808). The secondary antibodies against the above primary antibodies were: Alexa Fluor 647‐conjugated anti‐mouse goat IgG (H+L) (1:1000, Invitrogen, A‐21236), Alexa Fluor 488‐conjugated anti‐rat goat IgG (H+L) (1:1000, Invitrogen, A‐11006), and Alexa Fluor 568‐conjugated anti‐rabbit goat IgG (H+L) (1:1000, Invitrogen, A‐11036). Alexa Fluor 568‐conjugated Streptavidin (1:1000, Invitrogen, S11226) was utilized to stain biotinylated biomolecules.

### 4.13 Immunoblotting Analysis of Biotinylated Proteins

Following APEX labeling, cells were lysed for 15 min at 4°C in RIPA buffer (25 mM Tris‐HCl, pH 7.6, 150 mM NaCl, 1% NP‐40, 1% sodium deoxycholate, 2% SDS) supplemented with protease inhibitors. The lysate was sonicated on ice, centrifuged at 20 000 g for 10 min at 4°C, mixed with 5× loading buffer, and then heated at 95°C for 10 min before Western blot analysis.

Proteins were separated by SDS‐PAGE and transferred to a PVDF membrane. The membrane was blocked with 3% BSA in TBST for 1 h at room temperature. For detection of biotinylated proteins, the blot was probed with 0.2 µg/mL streptavidin‐HRP for 1 h at room temperature. To detect V5 tag or α‐tubulin, membranes were incubated with mouse anti‐V5 (1:5000) or mouse anti‐α‐tubulin (1:5000) primary antibodies, followed by HRP‐conjugated goat anti‐mouse IgG (1:5000). All antibody incubations were followed by three washes with TBST. Chemiluminescence signals were acquired using a ChemiDoc imaging system.

Blot images were analyzed using Fiji [46]. Following background subtraction, the intensities of anti‐V5 (Figures S5B and S14B) or biotinylation (Figure S6) signals were quantified and subsequently normalized to their corresponding α‐tubulin signal to account for loading differences.

### 4.14 Affinity Purification of Biotinylated RNAs

Following ^L242F^APEX2‐mediated labeling, cells were lysed by the addition of TRIzol reagent, and total RNAs were extracted according to the manufacturer's protocol. The extracted RNAs were then treated with DNase I at 37°C for 30 min to remove any contaminating genomic DNA, followed by purification using the RNA Clean & Concentrator kit. A 10 µL aliquot of purified RNA was reserved as the Input sample.

The enrichment of biotinylated RNAs was performed following a previously reported protocol [8]. Briefly, Dynabeads MyOne Streptavidin C1 beads (using 10 µL beads per 50 µg of RNA) were washed three times with bead washing buffer (5 mM Tris, pH 7.5, 1 M NaCl, 0.5 mM EDTA, 0.1% v/v Tween‐20), followed by two washes in Solution A (0.1 M NaOH and 0.05 M NaCl) and one wash in Solution B (0.1 M NaCl). The beads were then blocked by incubating with bead washing buffer supplemented with 1 mg/mL BSA and 1 mg/mL yeast tRNA at room temperature for 2 h with agitation. Subsequently, the beads were washed three times with bead washing buffer, followed by incubation with purified RNAs in bead washing buffer supplemented with 1 U/µL RiboLock RNase Inhibitor at room temperature for 40–50 min with thorough mixing. The biotinylated RNA‐bound beads were washed three times with bead washing buffer, twice with urea buffer (4 M urea and 0.1% SDS in PBS), and twice with PBS at room temperature to remove non‐specific adsorption. Finally, biotinylated RNAs were eluted with RNA elution buffer (95% formamide, 10 mM EDTA, and 1.5 mM D‐biotin) at 50°C for 5 min, followed by 90°C for 5 min with agitation.

The eluted RNAs were then purified with TRIzol reagent according to the manufacturer's instructions. To aid precipitation, 20 µg of glycogen was added to the aqueous phase before performing isopropanol precipitation. The purified biotinylated RNAs were dissolved in 20 µL of RNase‐free water and termed as the Enrich sample for labeled samples and the Control sample for negative controls omitting biotin probes or H_2_O_2_.

### 4.15 RT‐qPCR Analysis of Biotinylated RNAs

For RT‐qPCR quantification of RNAs captured by APEX labeling, 1 µg of Input, 6 µL of Enrich, and 6 µL of Control samples were reverse transcribed following the Protoscript II First Strand cDNA Synthesis Kit protocol using random primers. The synthesized cDNAs were used as templates for qPCR with Power Up SYBR Green Master Mix, using the primers listed in Table S2. Each gene was measured in four technical replicates.

The percent recovery in this study for a specific RNA species in the RT‐qPCR analysis was calculated as follows:

1. In the total RNA prior to streptavidin pulldown, the Ct (cycle threshold) value for a specific RNA was measured as Ct_Input;
2. In RNAs recovered after affinity purification, the Ct value was measured as Ct_Enrich;
3. The difference in Ct values was calculated as: ΔCt=Ct_Enrich−Ct_Input;
4. The percent recovery was then calculated as:
(1)

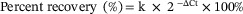

Where k is a scaling factor that accounts for the amounts of RNA subjected to streptavidin enrichment, RNA template used for reverse transcription, and cDNA template for qPCR. The scaling factor k was calculated as follows:

(2) $$k=\frac{b}{a}\;\times \;\frac{x}{y}\;\times \;\frac{m}{n}$$

Where:

a—mass (µg) of total RNA for affinity purification

b—mass (µg) of total RNA used for cDNA_Input preparation

x—Volume (µL) of RNase‐free water used to dissolve eluted RNA

y—volume (µL) of eluted RNA used for cDNA_Enrich preparation

m—volume (µL) of cDNA_Input used for qPCR

n—volume (µL) of cDNA_Enrich used for qPCR

Enrichment was determined as the percent recovery of target genes relative to off‐target (negative control) genes.

### 4.16 Drug Perturbation Assay

HEK293T cells cultured on Matrigel‐coated coverslips were treated with the indicated drugs at the following final concentration: 0.2% DMSO, 100 µg/mL puromycin, 200 µg/mL cycloheximide, 2 µg/mL harringtonine, or 25 nM leptomycin B. All drug treatments were performed for 20 min, except for leptomycin B, which was applied for 2 h. After treatment, cells were washed with PBS once, followed by fixation for subsequent immunofluorescence or smFISH imaging.

### 4.17 EGFP Reporter RNA Assay

HEK293T cells were transfected with the indicated constructs using Lipofectamine 3000 transfection reagent according to the manufacturer's instructions. Briefly, approximately 100 000 cells were seeded in a 24‐well plate. When reaching 80–90% confluence, cells were transfected with 0.5 µg plasmid DNA using 1 µL Lipofectamine 3000 transfection reagent in 50 µL OPTI‐MEM. After 6 h, the medium was replaced with fresh complete medium. At 18–24 h post transfection, cells were trypsinized and replated on Matrigel‐coated coverslips. Following an additional 18–24 h culture, cells were washed with PBS and fixed for subsequent smFISH imaging.

### 4.18 Single‐Molecule RNA FISH Imaging

smFISH probes targeting endogenous mRNA or the EGFP open reading frame were designed using Oligostan [47]. Each primary probe consisted of a gene‐specific region complementary to the target RNA, flanked by universal Flap X or Flap Y overhangs. Primary probes were synthesized by Genewiz (Tianjin, China), and dissolved in TE buffer (pH 8.0) to a stock concentration of 100 µM. For each target RNA, an equimolar mixture of all 20‐30 corresponding primary probes was prepared, and then diluted fivefold with TE buffer. Alexa Fluor 568‐ or Alexa Fluor 647‐conjugated FLAP probes were synthesized by Invitrogen (Shanghai, China). The lyophilized fluorescent probes were resuspended in TE buffer to a final concentration of 10 µM. The sequences of all primary probes and FLAP probes used in this study are listed in Data S3.

For smFISH imaging, cells were fixed in 3.2% paraformaldehyde (PFA) dissolved in PBSM (PBS with 1 mM MgCl_2_) at room temperature for 10 min. After fixation, cells were washed three times with ice‐cold PBSM containing 10 mM glycine, and then permeabilized at 4°C for 20 min in PBSM supplemented with 0.1% Triton X‐100 and 2 mM vanadyl ribonucleoside complex (VRC). Following two washes with PBSM, cells were equilibrated in prehyb‐30 buffer (30% formamide, 2× SSC) at room temperature for 10 min with gentle agitation. Primary probe hybridization was performed overnight at 37 °C in hybridization buffer containing 10% dextran sulfate, 30% formamide, 2× SSC, 2 mM VRC, 10 µg/ml salmon sperm DNA, 10 µg/ml *E. coli* tRNA, 10 µg/ml BSA, and 100 nM primary probe mix.

After hybridization, cells were washed three times with prehyb‐30 buffer at 37°C and twice with 2× SSC at room temperature. Then, cells were equilibrated with prehyb‐10 buffer (10% formamide, 2× SSC) at 37°C for 10 min, followed by incubation in secondary probe hybridization buffer (10% dextran sulfate, 10% formamide, 2× SSC, 2 mM VRC, 10 µg/ml salmon sperm DNA, 10 µg/ml *E. coli* tRNA, 10 µg/ml BSA, and 10 nM FLAP probes) at 37°C for 3 h. Subsequently, cells were washed twice with prehyb‐10 buffer at 37°C, once with 2× SSC buffer, and once with PBSM at room temperature. Following the final wash, cells were incubated with anti‐MKLP1 antibody and/or anti‐α‐tubulin antibody diluted in PBSM at 4°C overnight. After washing with PBSM three times, cells were incubated with corresponding secondary antibodies diluted in PBSM. Finally, cells were washed with PBSM, counterstained with DAPI, and mounted in Fluoromount‐G anti‐fade mounting medium for imaging.

### 4.19 Image Acquiring and Analysis

Immunofluorescence and smFISH imaging were performed on an inverted fluorescence microscope (Nikon‐TiE) equipped with a spinning disk confocal unit (Yokogawa CSU‐X1) and a scientific CMOS camera (Hamamatsu ORCA‐Flash 4.0 v2). Images were collected using a 40× (NA 1.3) or 60× (NA 1.4) oil‐immersion objective, with microscope control and image acquisition managed by µManager software [48]. Laser power for fluorescence excitation ranged from 1 to 6 W/cm^2^. For smFISH imaging, a 60× oil‐immersion objective lens was used with an intermediate magnification switch (1.5×) engaged to enhance spatial resolution. Additionally, images were collected as *z*‐stacks with one plane every 0.5 µm. All fluorescence images were processed and analyzed using Fiji.

For nucleocytoplasmic distribution analysis in Figure S10B, cells and nuclei were segmented using Cellpose3 [49] based on EGFP and DAPI signals, respectively. After background subtraction, the integrated fluorescence intensities of whole‐cell EGFP and nuclear EGFP were measured in Fiji. Cytoplasmic EGFP intensity was obtained by subtracting nuclear intensity from whole‐cell intensity. Mean cytoplasmic and nuclear intensities were then calculated by dividing each integrated intensity by the corresponding area. Finally, the cytoplasmic‐to‐nuclear (Cyto/Nuc) ratio for an individual cell was derived from these values.

For Pearson correlation analysis in Figure S11E, cells were segmented based on the anti‐V5 signal using Cellpose3. Following background subtraction, Pearson's correlation coefficient *r* between the anti‐V5 and biotinylation signals was calculated for individual cell using the EzColocalization plugin [50] in Fiji.

For smFISH imaging analysis, mitotic cells were manually identified based on condensed DNA morphology. Specific mitotic phases (metaphase, anaphase, telophase, and cytokinesis) were further classified according to spindle morphology and the presence or absence of the midbody. RNA spots were detected, localized, and quantified using the RS‐FISH [51] plugin in Fiji. Briefly, z‐stack images were maximum‐intensity projected. Cell boundaries were manually delineated based on DAPI or anti‐α‐tubulin signals, and midbody regions were defined using anti‐KIF23 staining or ANLN‐EGFP fluorescence. Detected RNA spots were assigned as midbody‐localized if they fell within the midbody mask, implemented via the Mask Filtering plugin of RS‐FISH. The proportion of midbody‐localized RNAs was determined by calculating the ratio of RNA spots within the midbody region to the total number of spots detected in the same cell. Statistical significance of differences in localization was assessed using a two‐sided Mann–Whitney test. For drug perturbation and EGFP reporter assays, only cells in telophase or cytokinesis (identified by DAPI or α‐tubulin staining) were included in the quantitative analysis.

### 4.20 Preparation of cDNA Library for Next Generation Sequencing

For cDNA library preparation, 1 µg of Input, 14 µL of Enrich, and 14 µL of Control samples from each biological replicate experiment were subjected to library construction using the NEBNext Ultra II RNA Library Prep Kit for Illumina with the Poly(A) mRNA Magnetic Isolation Module, following the manufacturer's instructions. Briefly, after isolating poly(A)+ RNA using magnetic oligo(dT) beads, the RNAs were fragmented at 94°C for 10 min. The fragmented RNAs were then subjected to first‐strand cDNA synthesis, second‐strand cDNA synthesis, end preparation, adaptor ligation, and PCR amplification. The number of PCR cycles for Input, Enrich, and Control samples were 10, 14‐18, and 20‐22, respectively.

Two rounds of size fractionation were performed to obtain cDNA segments distributed between 300 and 400 bp using 0.7× and 0.2× VAHTS DNA Clean Beads. The size distribution was further analyzed on the Fragment Analyzer. Samples with good quality were subjected to deep‐sequencing for 150 bp paired reads on the Illumina HiSeq X Ten platform.

### 4.21 NGS Data Analysis

The sequencing reads for each library were aligned against the human genome assembly GRCh38 (hg38), downloaded from the Ensembl project, using HISAT2 (v2.1.0) [52]. Subsequently, the results of alignments were sorted by read names. The read counts of each gene were measured with the matching gene annotation (v.87) from Ensembl by HTSeq v0.6.1, utilizing the option “‐stranded no” [53]. The FPKM (Fragments Per Kilobase of transcript per Million mapped reads) of each gene was calculated based on raw counts to estimate the reproducibility between biological replicates.

To identify ERM‐localized transcripts, DESeq2 [34] analysis was performed on ERM‐labeled samples versus three negative controls: (1) total RNAs extracted from labeled cells (ERM_Input); (2) RNAs enriched from unlabeled samples omitting the Btn‐An probe (ERM_Control); (3) RNAs labeled by cytoplasm‐localized ^L242F^APEX2 (NES_Enrich). To enhance the quality of the analysis, genes with low abundance (average raw counts < 100) were filtered out. Receiver Operating Characteristic (ROC) analysis was employed to evaluate the sensitivity and specificity of different DESeq2 protocols. True positives were defined as ER‐enriched mRNAs identified using proximity‐specific ribosome profiling [16], while false positives were defined as mRNAs encoding non‐secretory proteins annotated in a previous study [54]. Only genes with FDR‐adjusted *p*‐values less than 0.05 were subjected to ROC analysis. The comparison ERM_Enrich vs. NES_Enrich yielded the highest area under the curve (AUC), supporting its selection for downstream analysis. The optimal fold‐change cut‐off was determined as the value that maximized the difference between the true positive rate and false positive rate. The ER membrane‐localized transcripts captured by APEX2 or ^L242F^APEX2 were summarized in Data S1.

The secretome RNA was defined based on GOCC and HPA databases. GOCC‐secretome was generated according to previous studies [13, 18, 20]. Briefly, GOCC‐secretome was defined as the ensemble of genes whose GOCC annotations include compartments linked to the secretory pathway, including ER, Golgi apparatus, endosome, lysosome, peroxisome, vesicles, plasma membrane, and extracellular region. The specific GO terms utilized in our study was listed in Table S3. HPA‐secretome was defined using annotations from the HPA database [21], including terms such as ‘Endoplasmic Reticulum’, ‘Golgi Apparatus’, ‘Plasma Membrane’, ‘Secreted proteins’, and ‘Vesicles’.

To identify the midbody‐proximal transcriptome, DESeq2 analysis was performed by comparing RNAs enriched from KIF23‐^L242F^APEX2 or BIRC5‐^L242F^‐APEX2 labeled samples against those from control samples omitting the H_2_O_2_ trigger. Genes with an average read count below 100 were excluded from further analysis. Applying a threshold of fold‐change > 1.5 and FDR‐adjusted *p*‐value < 0.05, we identified 1530 and 863 enriched RNAs from the two respective datasets. The intersection of these two lists yielded 767 RNAs, which were defined as the midbody‐proximal transcriptome in this study (Data S2).

To investigate whether these RNAs likely undergo local translation, we compiled a separate list of genes encoding proteins annotated to localize at the midbody, termed midbody‐related mRNAs. Protein localizations were defined based on annotations from the HPA database (‘midbody ring’, ‘midbody’, ‘cytokinetic bridge’, ‘cleavage furrow’) and Uniprot resource (Subcellular location: ‘cleavage furrow’, ‘midbody’, ‘midbody ring’). For HPA annotations, only entries with reliability scores of ‘Approved’, ‘Supported’, or ‘Enhanced’ were included in this list. This process generated a final list of 333 midbody‐related mRNAs, provided in Data S2.

GO enrichment analysis for GOCC and COBP terms was conducted using the PANTHER Over‐representation Test with Fisher's exact test [55]. Only terms with an FDR‐corrected *p‐*value < 0.05 were considered significantly enriched. All graphs summarizing the RNA‐seq analysis were generated using Origin 2023b.

## Author Contributions

P.Z. conceived the project. G.W., Y.L., and P.Z. designed experiments. G.W., Y.L., and P.M. performed experiments. G.W., Y.L., P.M., and P.Z. analyzed the data and wrote the paper.

## Conflicts of Interest

The authors declare no conflicts of interest.

## Supporting information




**Supporting file 1**: advs75012‐sup‐0001‐SuppMat.pdf

**Supporting file 2**: advs75012‐sup‐0002‐Data.zip

## Data Availability

All data other than RNA sequencing data are incorporated into the article and its Supplementary Material. The sequencing data underlying this article have been deposited in the National Center for Biotechnology Information Gene Expression Omnibus (accession code: GSE262170)
