# Supplementary material for: Directed Evolution Improves the Catalytic Efficiency of APEX2‐Mediated Proximity‐Dependent RNA Labeling
Source: Adv Sci (Weinh). 2026 Mar 28;13(32):e75012. doi: 10.1002/advs.75012 (PMC13252616; doi:10.1002/advs.75012)
Supplement: Supplementary file 1 — Supporting file 1: advs75012‐sup‐0001‐SuppMat.pdf [file ADVS-13-e75012-s001.pdf]

Supplementary Information

Table of contents

Supplementary Figures ... 1
- Figure S1 ... 1
- Figure S2 ... 2
- Figure S3 ... 3
- Figure S4 ... 3
- Figure S5 ... 4
- Figure S6 ... 4
- Figure S7 ... 5
- Figure S8 ... 6
- Figure S9 ... 7
- Figure S10 ... 8
- Figure S11 ... 9
- Figure S12 ... 10
- Figure S13 ... 10
- Figure S14 ... 11
- Figure S15 ... 11
- Figure S16 ... 12
- Figure S17 ... 13
- Figure S18 ... 14
- Figure S19 ... 15
- Figure S20 ... 16
- Figure S21 ... 17
- Figure S22 ... 18
- Figure S23 ... 19
- Figure S24 ... 20
- Figure S25 ... 21
- Figure S26 ... 22
- Figure S27 ... 23
- Figure S28 ... 23
- Figure S29 ... 24

Supplementary Tables ... 25
- Table S1. Key resources table used in this study ... 25

Table S2. qPCR primers used in this study 32
Table S3. List of GO terms used to define GOCC-secretome 33
Table S4. gRNA sequences and genotyping primers 34
Table S5. EGFP reporter plasmids used in this study 35

References 36

Supplementary Figures

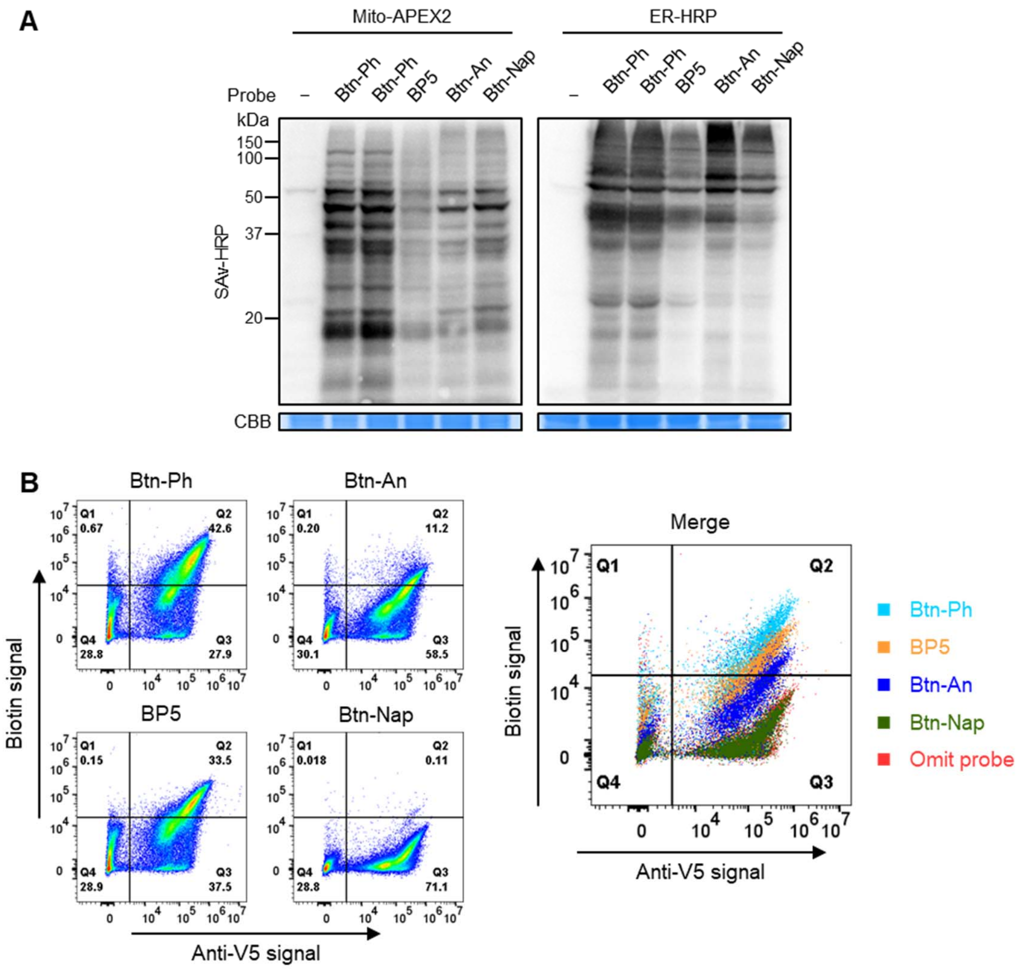

Figure S1. Evaluation of high-activity probes in mammalian cells and on the yeast surface. (**A**) HEK293T cells stably expressing either mitochondrial matrix-targeted APEX2 (left panels) or ER lumen-targeted HRP (right panels) were labeled with 500 μM of various aromatic substrates. Biotinylated proteins were detected by streptavidin blotting (upper panels). Coomassie Brilliant Blue (CBB) staining of total protein serves as a loading control (lower panels). (**B**) Yeast surface display coupled with flow cytometry was used to profile substrate activity. EBY100 yeast displaying APEX2 were incubated with 100 μM of each substrate. Surface expression of APEX2 was monitored via anti-V5 staining, and biotinylation efficiency was quantified using fluorophore-conjugated streptavidin.

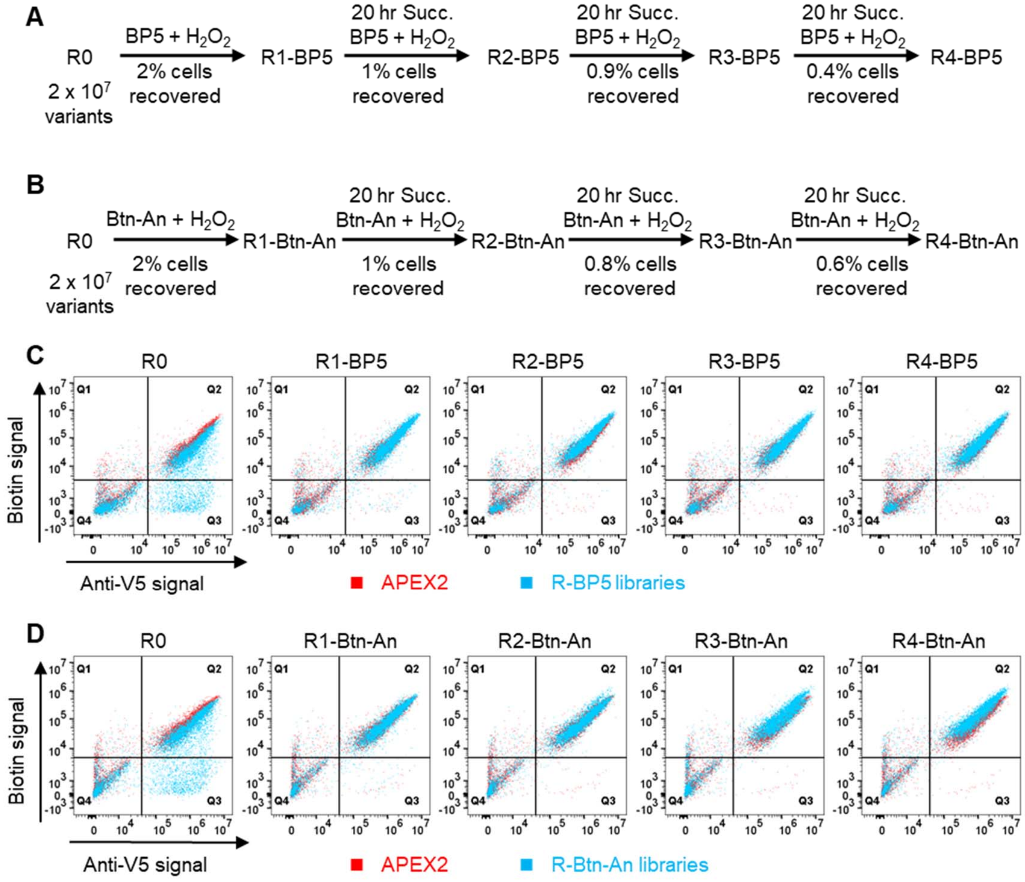

Figure S2. FACS-based screening of an APEX2 random mutagenesis library over four rounds. (**A**–**B**) Workflow for screening the APEX2 mutagenesis library with BP5 (**A**) or Btn-An (**B**) probe. Yeast cells displaying the library were labeled with 100 μM probe and 1 μM H_2O_2 for 1 min. Cells exhibiting a high biotinylation-to-V5 signal ratio were isolated by FACS. In subsequent rounds, 1 mM succinyl acetone (Succ.) was included during cell culture and peroxidase expression to impose selective pressure for efficient heme incorporation. (**C**–**D**) Flow cytometry profiles of the enriched populations from each round of the screening with BP5 **(C)** or Btn-An **(D)**. Surface expression of APEX2 variants was monitored via anti-V5 staining, and labeling activity was measured using fluorophore-conjugated streptavidin.

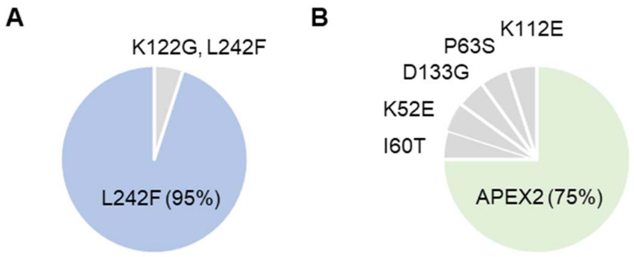

Figure S3. Mutation profile of clones isolated from the fourth-round (R4) libraries. Twenty individual clones from each enriched library (R4-Btn-An and R4-BP5) were isolated and subjected to Sanger sequencing. The bar plots depict the sequencing results for the R4-Btn-An **(A)** and R4-BP5 **(B)** libraries.

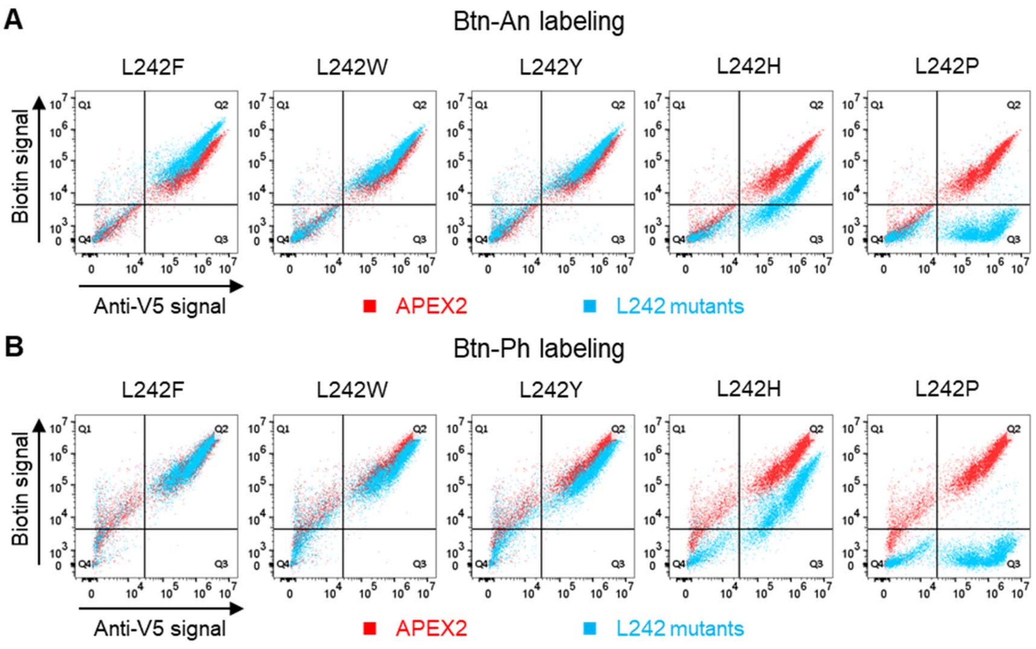

Figure S4. Flow cytometry comparison of labeling efficiency between APEX2 and its L242 mutants on the yeast surface. Yeast (EBY100) surface-displaying APEX2 or the indicated L242 mutants were labeled with 100 μM Btn-An (**A**) or Btn-Ph (**B**), followed by flow cytometry analysis. Surface expression of APEX2 variants was monitored via anti-V5 staining, and labeling activity was measured using fluorophore-conjugated streptavidin.

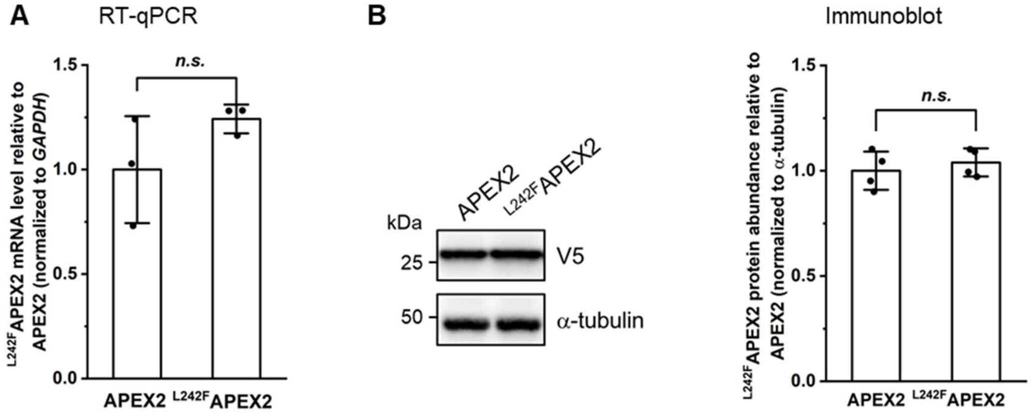

Figure S5. Expression levels of APEX2 variants in the mitochondrial matrix. Comparison of ^{L242F}APEX2 and APEX2 expression levels between mito-^{L242F}APEX2 and mito-APEX2 cell lines via RT-qPCR (**A**) and immunoblotting (**B**). (**A**) Box plot depicting relative RNA level normalized by *GAPDH*. Data are presented as mean ± SD of three biological replicates. (**B**) Left: Representative western blot of APEX2 variant expression. Right: Box plot showing relative protein level normalized by α-tubulin. Data are presented as mean ± SD of four biological replicates. For both analyses, the average value of APEX2 was set to 1.0. Statistical significance was determined using a two-sided *t* test. *n.s.*: not significant.

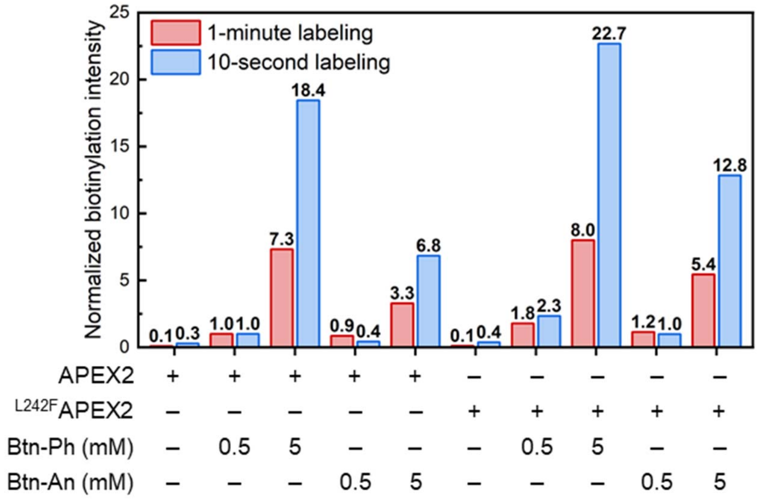

Figure S6. Quantification of biotinylation signals from immunoblots, related to Figure 2B and Figure S8A. Biotinylation signal intensities were quantified using Fiji, normalized to the corresponding α-tubulin signal to account for loading differences, and are presented as fold changes relative to the "APEX2 + 0.5 mM Btn-Ph" condition. The normalized fold change for each condition is indicated above the bar.

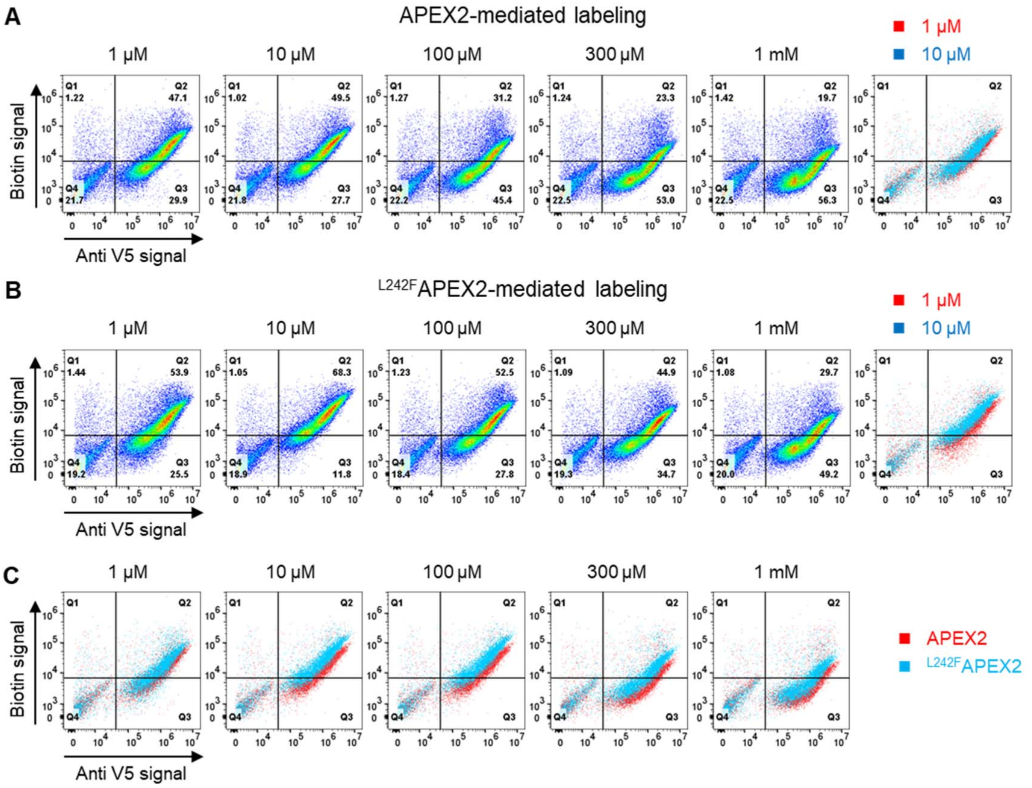

Figure S7. Titration of H_2O_2 concentration for APEX2- or $^{\mathrm{L242F}}$APEX2-mediated labeling on the yeast surface. Yeast (EBY100) cells displaying APEX2 (**A**) or $^{\mathrm{L242F}}$APEX2 (**B**) were labeled with Btn-An across a range of H_2O_2 concentrations. Surface expression of APEX2 variants was monitored via anti-V5 staining, and labeling activity was measured using fluorophore-conjugated streptavidin. The rightmost panels provide a direct comparison of labeling signals obtained with 1 μM versus 10 μM H_2O_2. (**C**) Direct comparison of the dose-response profiles from (**A**) and (**B**), overlaid to illustrate the differential H_2O_2 sensitivity of the two enzymes.

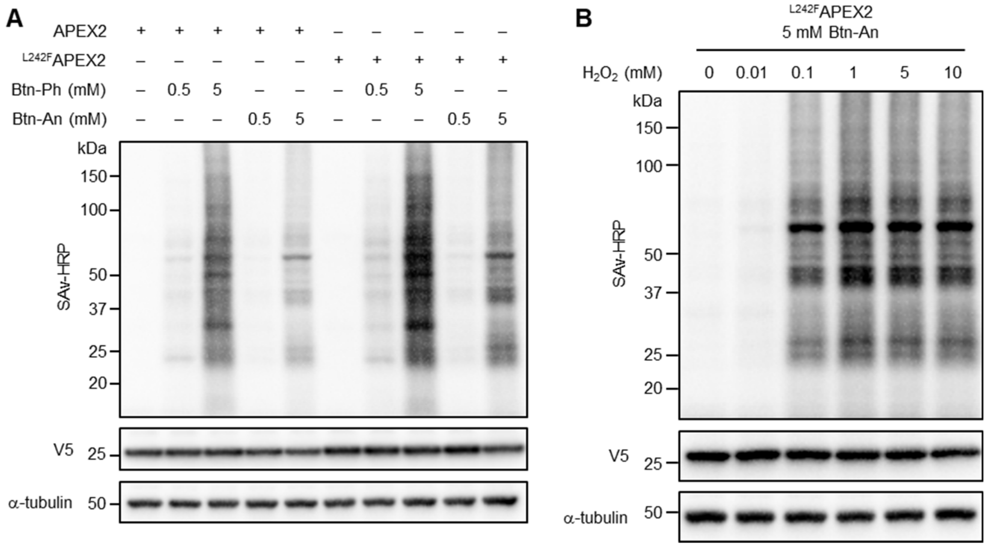

Figure S8. Effect of probe concentration and H_2O_2 dosage on APEX labeling under a 10-second labeling window. (**A**) Comparison of protein labeling efficiency catalyzed by either mitochondrial matrix-localized APEX2 or ^{L242F}APEX2 variant in the presence of Btn-Ph or Btn-An at a concentration of 0.5 mM or 5 mM. (**B**) Immunoblot analysis of labeling efficiency under varying H_2O_2 concentrations. Mito-^{L242F}APEX2 cells were incubated with 5 mM Btn-An for 30 minutes, followed by a 10-second stimulation with indicated concentrations of H_2O_2. Total biotinylated proteins were detected by streptavidin blotting (upper panel). Expression of APEX2 variants was verified by anti-V5 immunoblotting (middle panel). α-Tubulin was used as a loading control (lower panel).

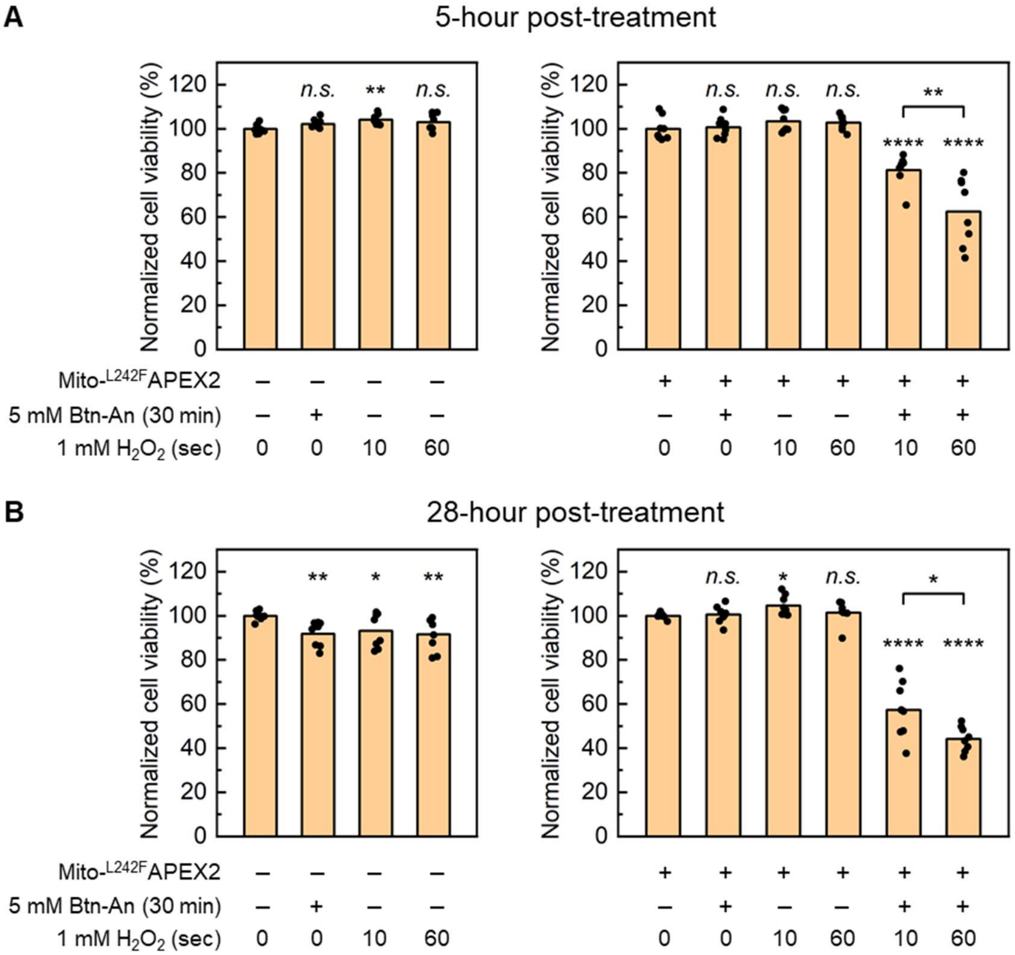

Figure S9. Cytotoxicity assessment of ^{L242F}APEX2-mediated labeling procedure via MTS assay. Cell viability of wild-type HEK293T (left panels) and mito-^{L242F}APEX2 cells (right panels) was assessed approximately 5 hours (1-hour recovery + 4-hour MTS incubation, **A**) and 28 hours (24-hour recovery + 4-hour MTS incubation, **B**) post indicated treatments. The cell viability at untreated condition was set as 100%. Data are the mean of 8 technical replicates from 2 independent biological replicates. Statistical significance was calculated by a two-sided *t* test. *n.s.*: not significant. *: $p < 0.05$. **: $p < 0.01$. ****: $p < 0.0001$.

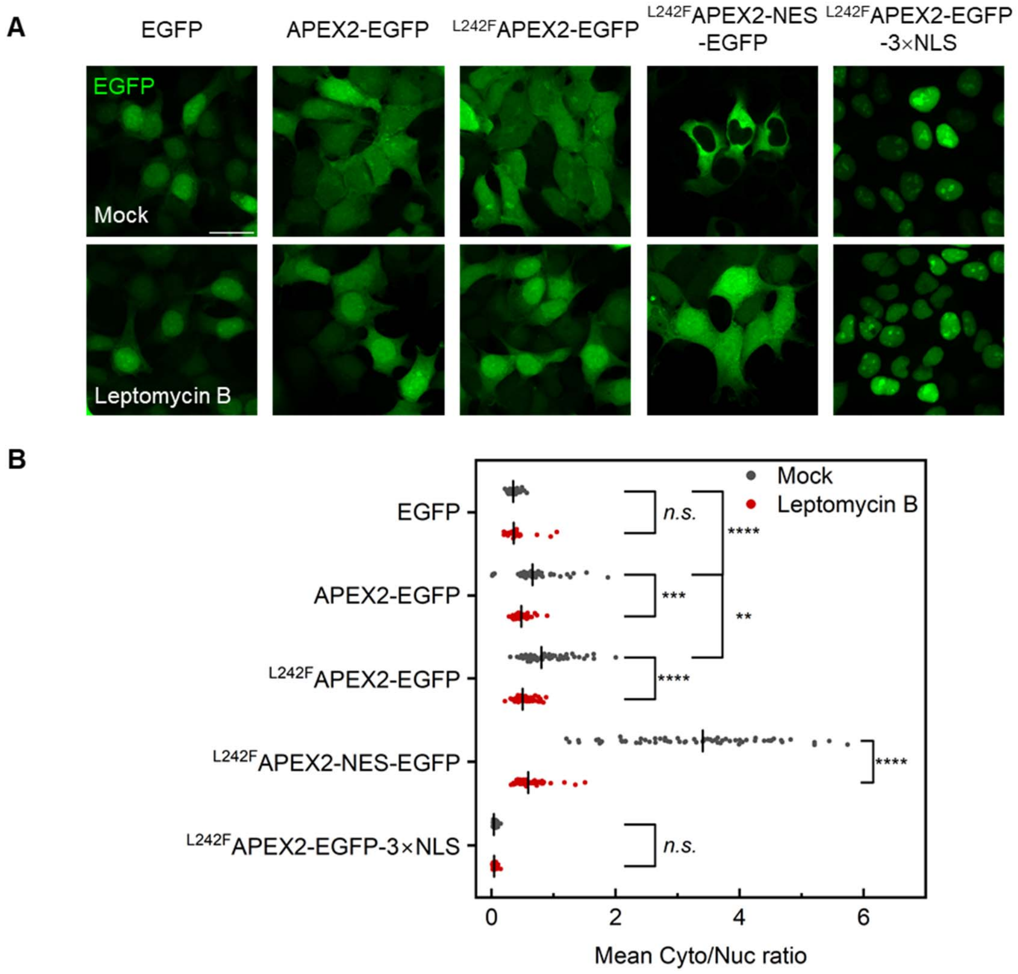

Figure S10. Assessment of nucleocytoplasmic distribution of APEX2 variants. (**A**) Representative fluorescence images show the localization of indicated EGFP fusion proteins. Cells were either untreated (mock; upper panels) or treated with leptomycin B (lower panels). Scale bar, 20 μm. (**B**) Quantification of cytosol-to-nucleus EGFP fluorescence intensity ratio for each construct. Data points were collected from at least 40 cells from 6–7 independent fields of view per condition. Statistical significance was determined by a two-sided *t* test. *n.s.*: not significant. **: $p < 0.01$. ***: $p < 0.001$. ****: $p < 0.0001$.

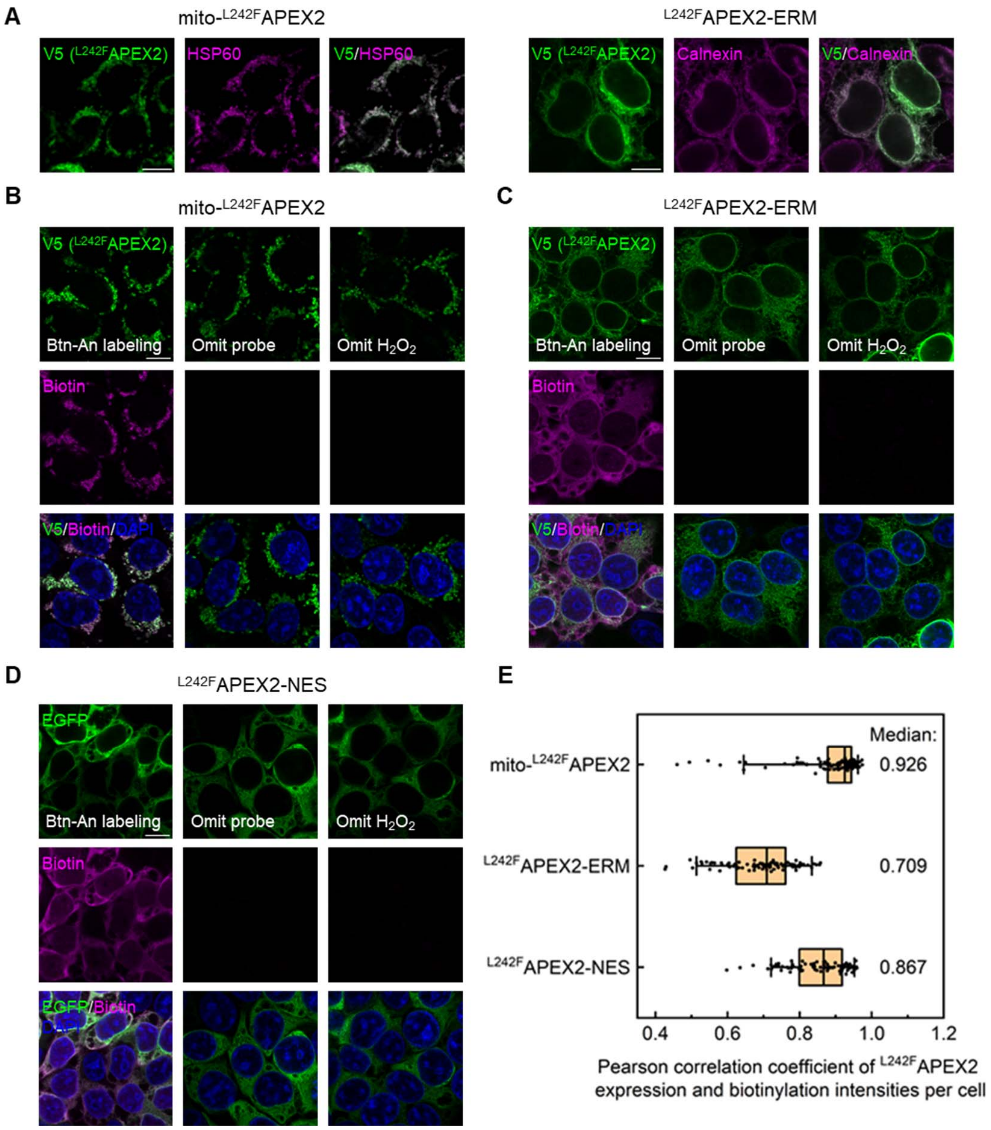

Figure S11. Immunofluorescence imaging of ^{L242F}APEX2-mediated labeling at distinct subcellular compartments. (**A**) Immunofluorescence images of HEK293T cells stably expressing mito-^{L242F}APEX2 or ^{L242F}APEX2-ERM. Green: ^{L242F}APEX2 expression; magenta: antibody staining against HSP60 (mitochondria) or Calnexin (ER); blue: DAPI. Scale bars,10 μm. (**B**–**D**) Representative immunofluorescence images illustrating the distribution of ^{L242F}APEX2 (green), biotinylation (magenta), and DAPI-stained nuclei (blue) in mito-^{L242F}APEX2 (**B**), ^{L242F}APEX2-ERM (**C**), and ^{L242F}APEX2-NES (**D**) cell lines. For each cell line, images correspond to: Btn-An labeled cells (left), a negative control omitting Btn-An (middle), and a negative control omitting H_2O_2 (right). Scale bar, 10 μm. (**E**) Quantification of colocalization between ^{L242F}APEX2 expression and biotinylation. The box plot shows the Pearson correlation coefficient *r* calculated for individual cells. A total of 112, 93, and 82 cells from 6–7 independent fields of view were analyzed for the three cell lines, respectively.

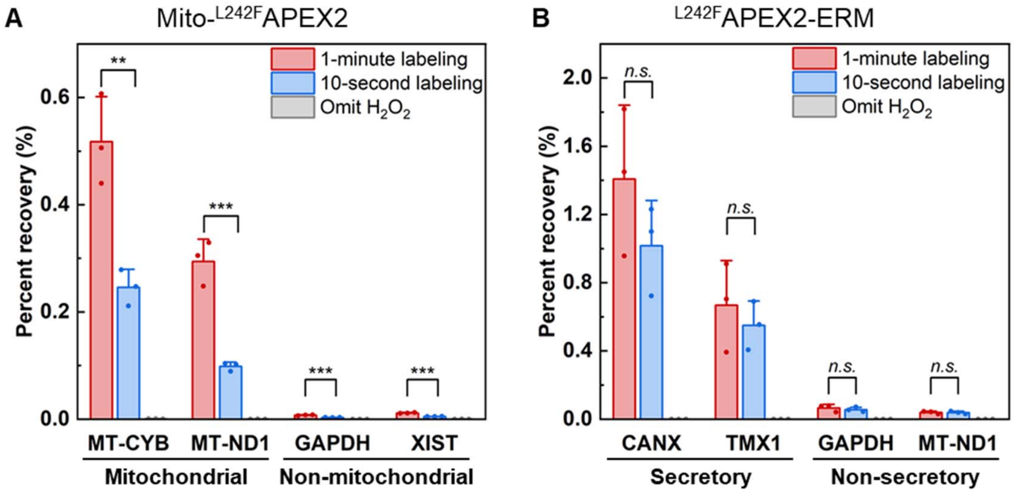

Figure S12. Comparison of RNA labeling efficiency between 1-minute and 10-second labeling durations. RT-qPCR analysis of RNA enrichment after L242FAPEX2-mediated labeling in the mitochondrial matrix **(A)** or ER membrane **(B)** using 5 mM Btn-An. Box plot depicts mean ± SD of three biological replicates. Statistical significance was calculated by a one-sided t test. *n.s.*: not significant. **: $p < 0.01$. ***: $p < 0.001$.

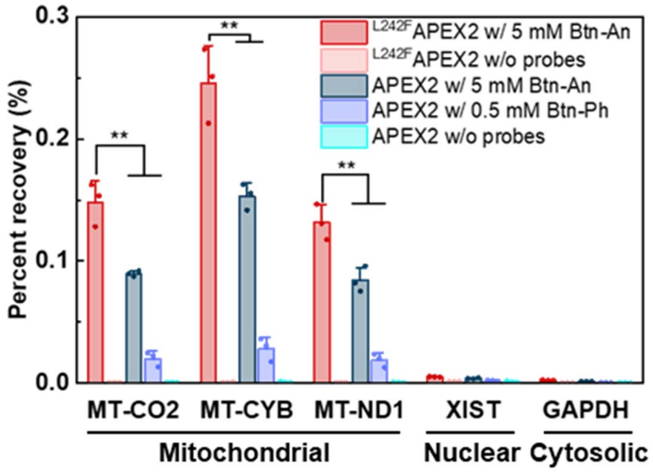

Figure S13. Comparison of RNA labeling efficiency between APEX2 and L242FAPEX2 in the mitochondrial matrix. RT-qPCR analysis of the recovery rates for RNAs labeled by APEX2 variants targeted to mitochondrial matrix following 10-second labeling. Data are presented as the mean ± SD of three biological replicates. Statistical significance was calculated by a one-sided t test. **: $p < 0.01$.

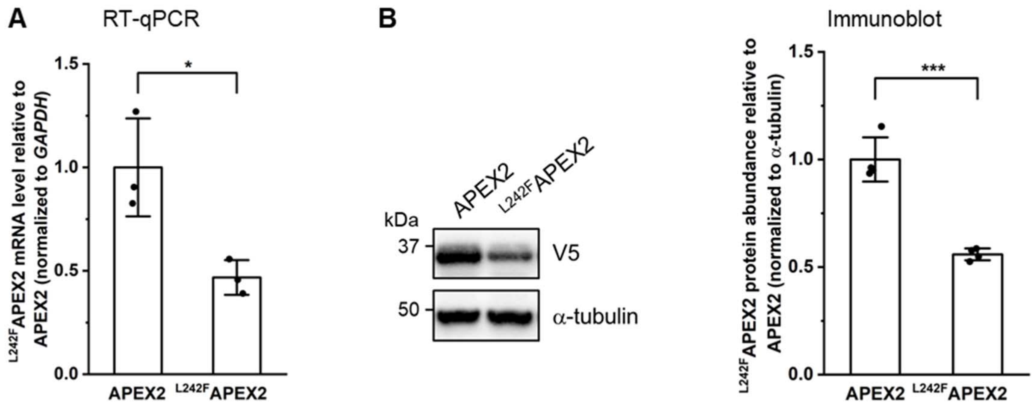

Figure S14. Expression levels of APEX2 variants in the ER membrane. Comparison of ^{L242F}APEX2 and APEX2 expression levels between ^{L242F}APEX2-ERM and APEX2-ERM cell lines via RT-qPCR (**A**) and immunoblotting (**B**). (**A**) Box plot depicting relative RNA level normalized by *GAPDH*. Data are presented as mean ± SD of three biological replicates. (**B**) Left: Representative western blot of APEX2 variant expression. Right: Box plot showing relative protein level normalized by α-tubulin. Data are presented as mean ± SD of four biological replicates. For both analyses, the average value of APEX2 was set to 1.0. Statistical significance was determined using a two-sided *t* test. *: $p < 0.05$. ***: $p < 0.001$.

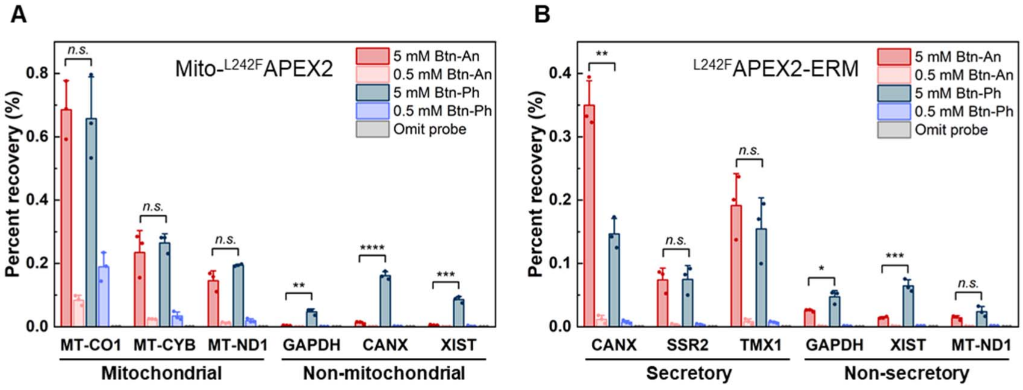

Figure S15. Comparison of RNA labeling efficiency between Btn-An and Btn-Ph probes catalyzed by ^{L242F}APEX2. RT-qPCR quantification of the RNA enrichment following a 10-second labeling window with 0.5 or 5 mM Btn-An or Btn-Ph, mediated by mitochondrial matrix-targeted (**A**) or ER membrane-targeted (**B**) ^{L242F}APEX2. Box plot depicts mean ± SD of three biological replicates. Statistical significance was calculated by a two-sided *t* test. *n.s.*: not significant. *: $p < 0.05$. **: $p < 0.01$. ***: $p < 0.001$. ****: $p < 0.0001$.

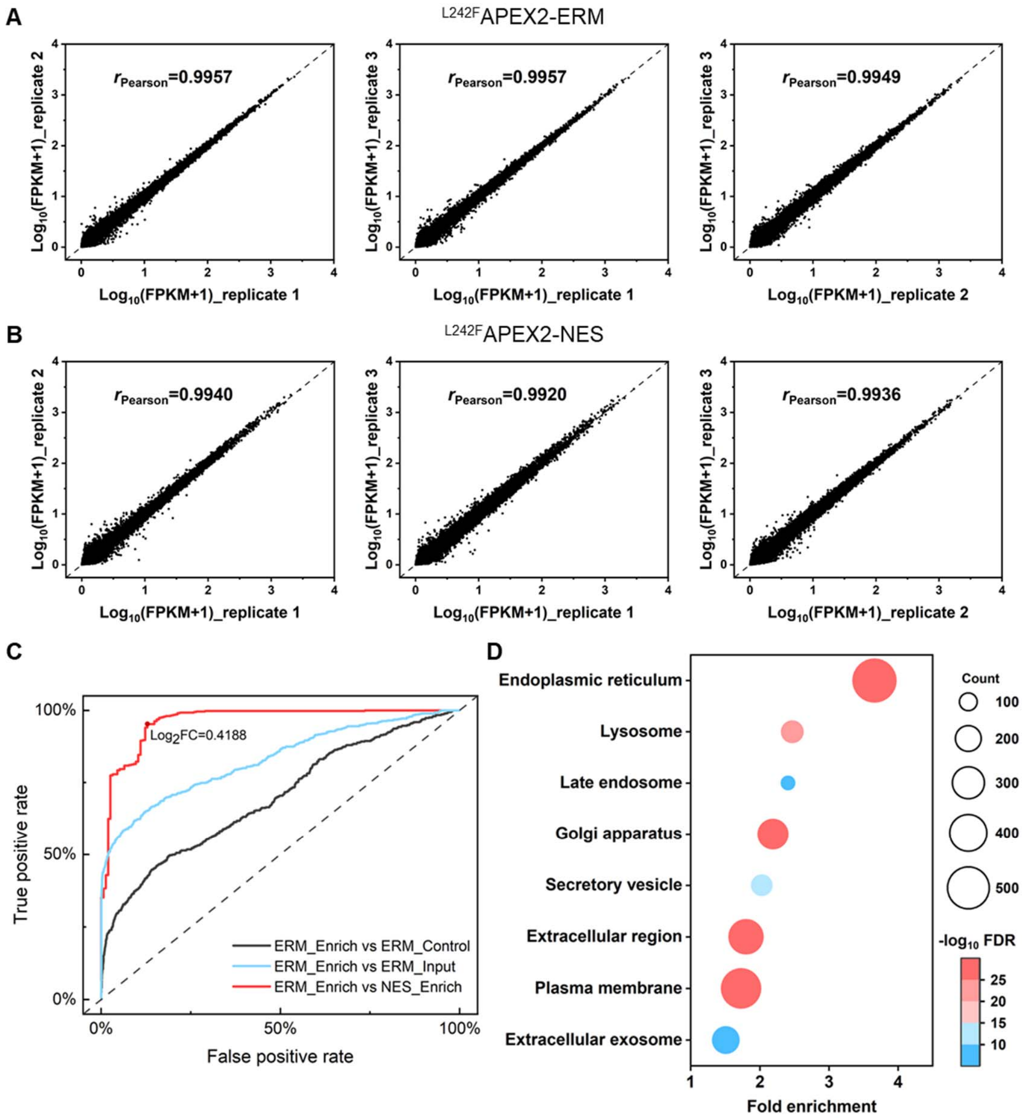

Figure S16. Transcriptome profiling at the ER membrane with ^{L242F}APEX2/Btn-An following 10-second labeling. (**A**–**B**) Scatter plots comparing RNA abundance between labeled samples from three independent biological replicates for the ^{L242F}APEX2-ERM (**A**) and ^{L242F}APEX2-NES (**B**) cell lines. (**C**) Receiver Operating Characteristic (ROC) curves evaluating the performance of ^{L242F}APEX2-mediated RNA labeling using different negative controls in DESeq2 analysis. The comparison ERM_Enrich vs NES_Enrich achieved the highest area under the curve (AUC). The value maximizing the difference between the true positive rate (TPR) and false positive rate (FPR) was set as the fold-change cutoff (Log_2FC=0.4188). (**D**) Bubble plot depicting Gene Ontology Cellular Component (GOCC) enrichment analysis of 1130 mRNAs captured by ^{L242F}APEX2. The circle size represents the number of genes per term, and color indicates the statistical significance.

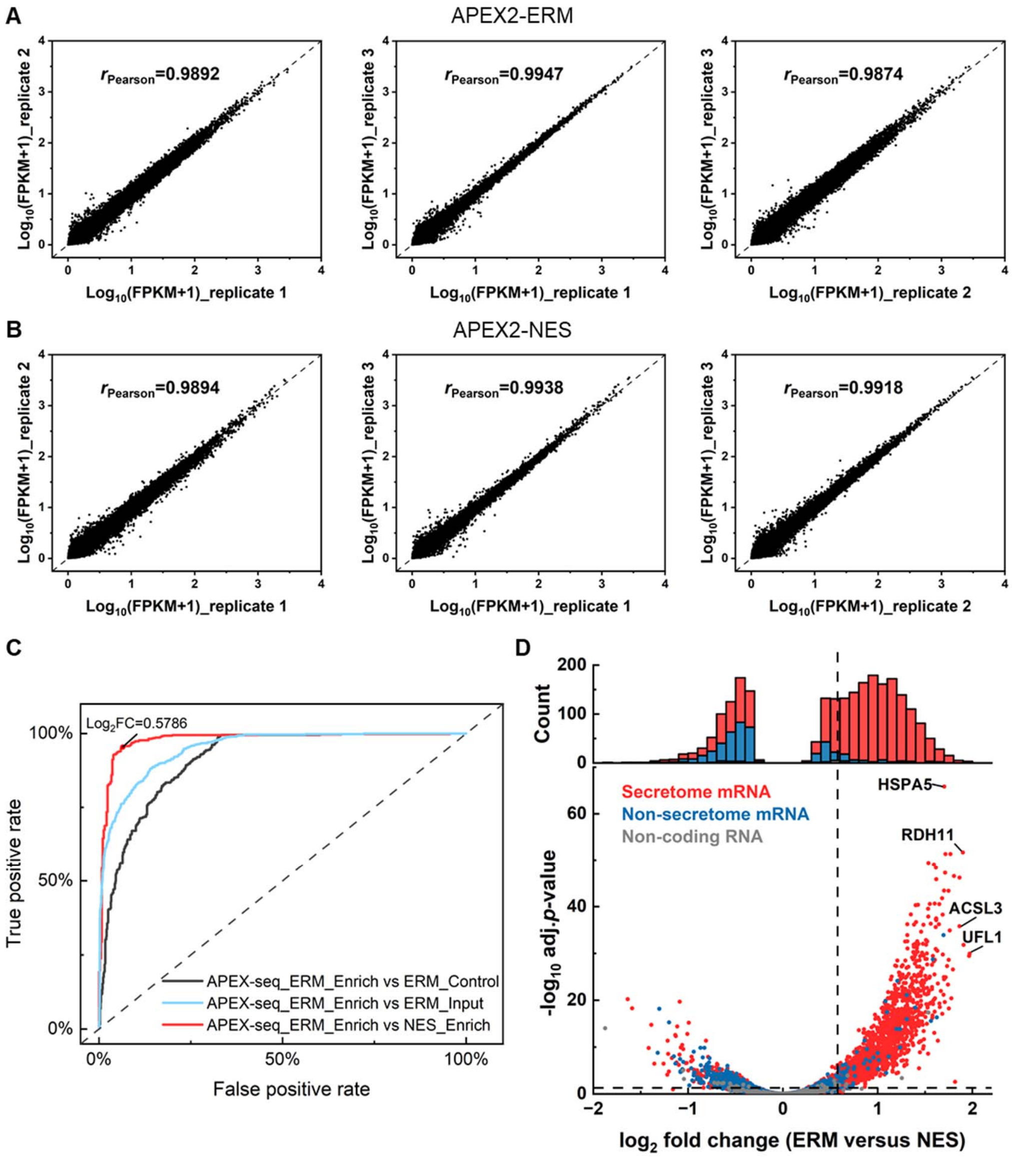

Figure S17. APEX-seq profiling of the ER membrane-localized transcriptome following 10-second labeling. (**A**-**B**) Scatter plots comparing RNA abundance between labeled samples from three independent biological replicates in the APEX2-ERM (**A**) and APEX2-NES (**B**) cell lines. (**C**) ROC curves evaluating the performance of different negative controls for APEX2-mediated RNA labeling in DESeq2 analysis. The comparison ERM_Enrich vs NES_Enrich achieved the highest AUC. The optimal $\log_2$ fold-change (0.5786) was determined as the value that maximized the difference between TPR and FPR. (**D**) Volcano plot presenting APEX2-mediated labeling of secretome mRNAs (red), non-secretome mRNAs (blue), and non-coding RNAs (grey). Horizontal dashed line indicates adj. p-value = 0.05. Vertical dashed line indicates the cutoff of $\log_2$ fold change (ERM versus NES) = 0.58. Upper panel, histograms showing the distribution of RNAs with adj. p-value < 0.05.

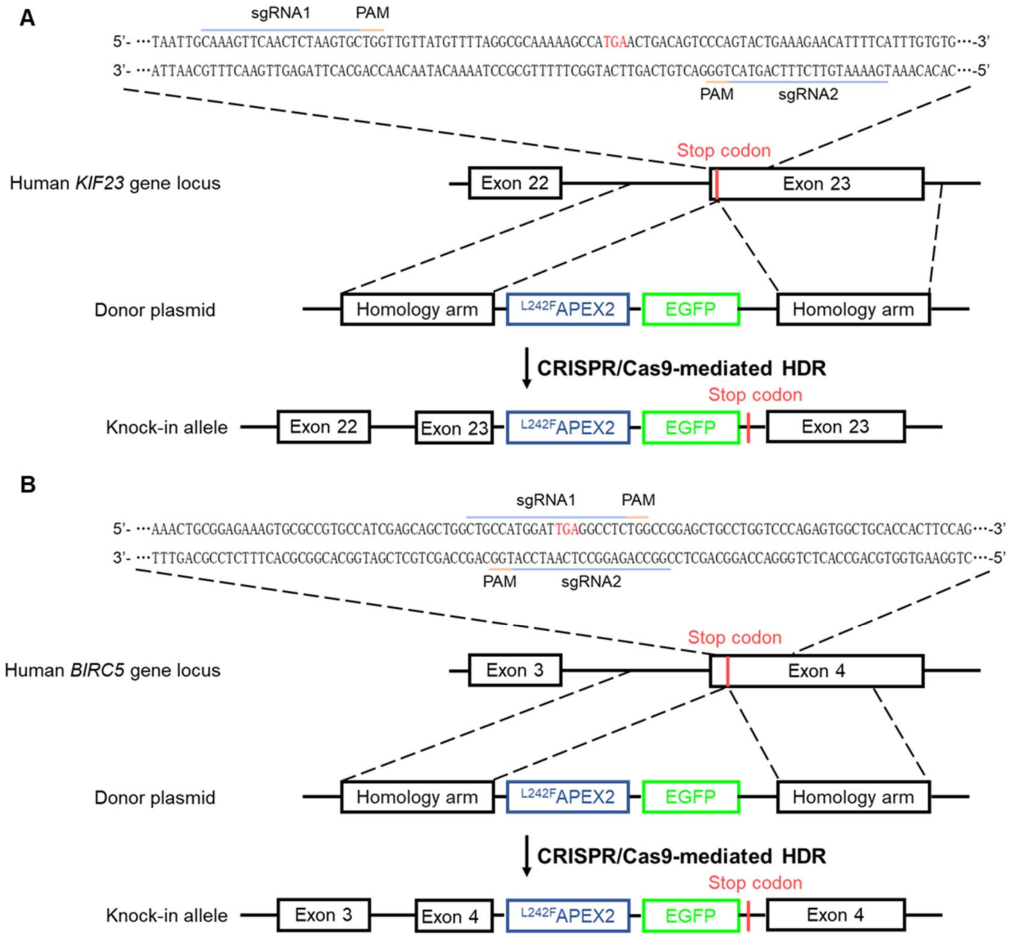

Figure S18. CRISPR/Cas9-mediated knock-in of ^{L242F}APEX2-EGFP at endogenous gene loci. Schematics illustrate the targeted integration of ^{L242F}APEX2-EGFP cassette immediately downstream of the stop codon in the *KIF23* (**A**) and *BIRC5* (**B**) gene loci. For each target, a pair of gRNAs direct Cas9 to generate double-strand breaks near the stop codon. Precise insertion of the cassette is achieved via homology-directed repair (HDR) using a donor DNA template.

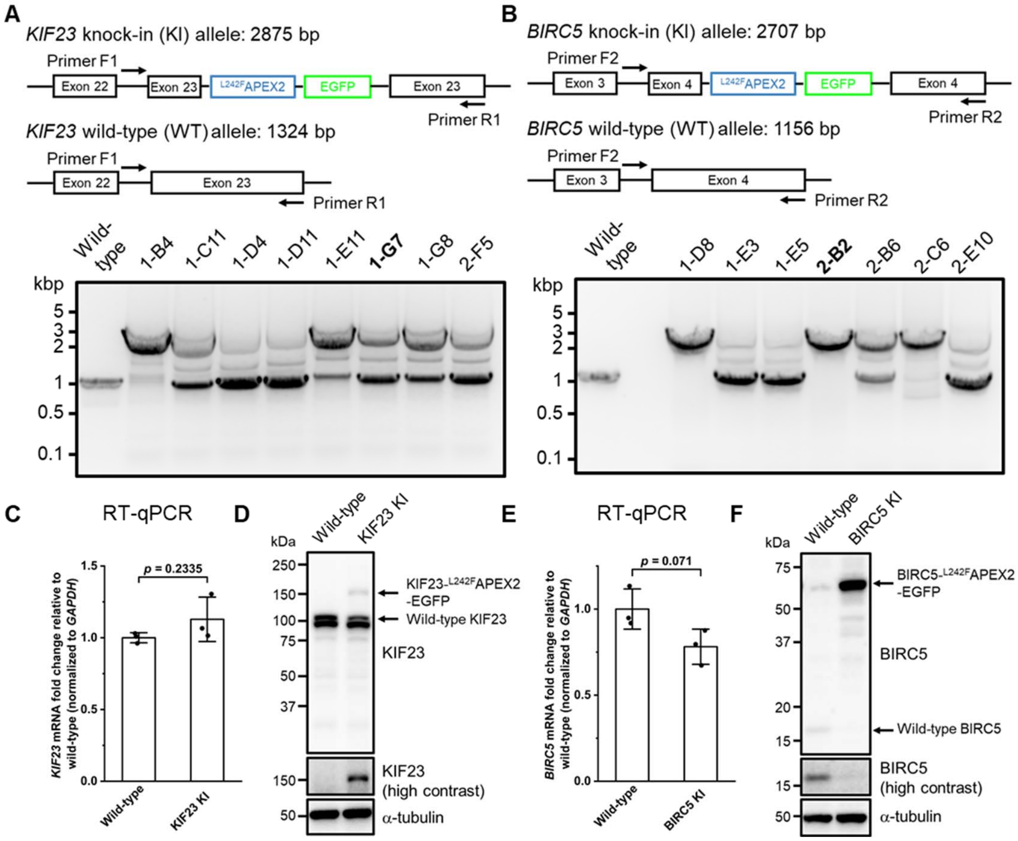

Figure S19. Characterization of single-cell clones with ^{L242F}APEX2 knocked in at the *KIF23* or *BIRC5* loci. (**A**–**B**) Genotyping of isolated EGFP-positive single-cell clones. Schematics (top) show the primer design for distinguishing wild-type and knock-in alleles. Agarose gel electrophoresis (bottom) of PCR products from genomic DNA of *KIF23* (**A**) or *BIRC5* (B) clones. The heterozygous clone KIF23 1-G7 and homozygous clone BIRC5 2-B2 were selected for downstream applications. (**C** and **E**) Comparison of *KIF23* (**C**) or *BIRC5* (**E**) transcript levels in wild-type versus knock-in cell lines via RT-qPCR. Data are presented as mean ± SD of three biological replicates. Expression in the wild-type cell line was set to 1.0. Statistical significance was determined using a two-sided *t* test. (**D** and **F**) Immunoblot analysis of KIF23 (**D**) or BIRC5 (**F**) protein expression levels in wild-type and knock-in cell lines. anti-KIF23 or anti-BIRC5 staining was used for detecting endogenous or edited proteins (upper panel). Middle panels show higher-contrast images to visualize specific bands. α-Tubulin served as a loading control (lower panel). Notably, the weaker anti-BIRC5 signal in wild-type lanes (**F**) may reflect lower transfer efficiency of this smaller protein, as comparable mRNA levels were detected (**E**).

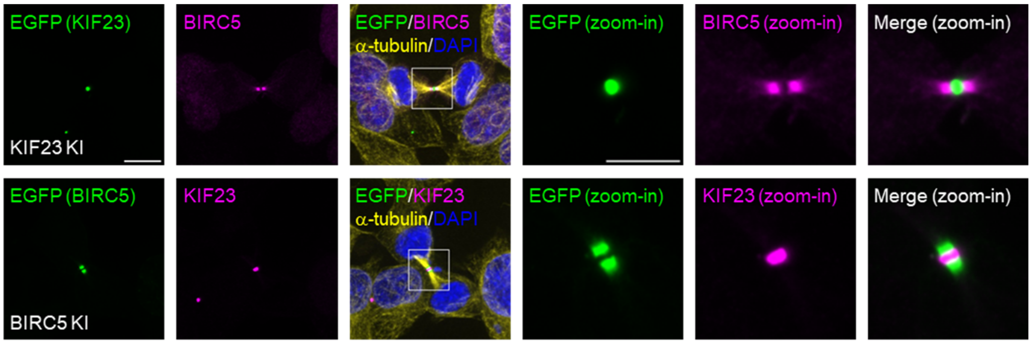

Figure S20. Validation of endogenously tagged ^{L242F}APEX2 localization in telophase cells. Representative immunofluorescence images of cells with ^{L242F}APEX2 knocked in at the *KIF23* (top row) or *BIRC5* (bottom row) locus. Green: ^{L242F}APEX2 expression; magenta: antibody staining against BIRC5 or KIF23; yellow: α-tubulin staining to visualize microtubules; blue: DAPI-stained nuclei. Scale bars: 10 μm (overview); 5 μm (zoom-in regions).

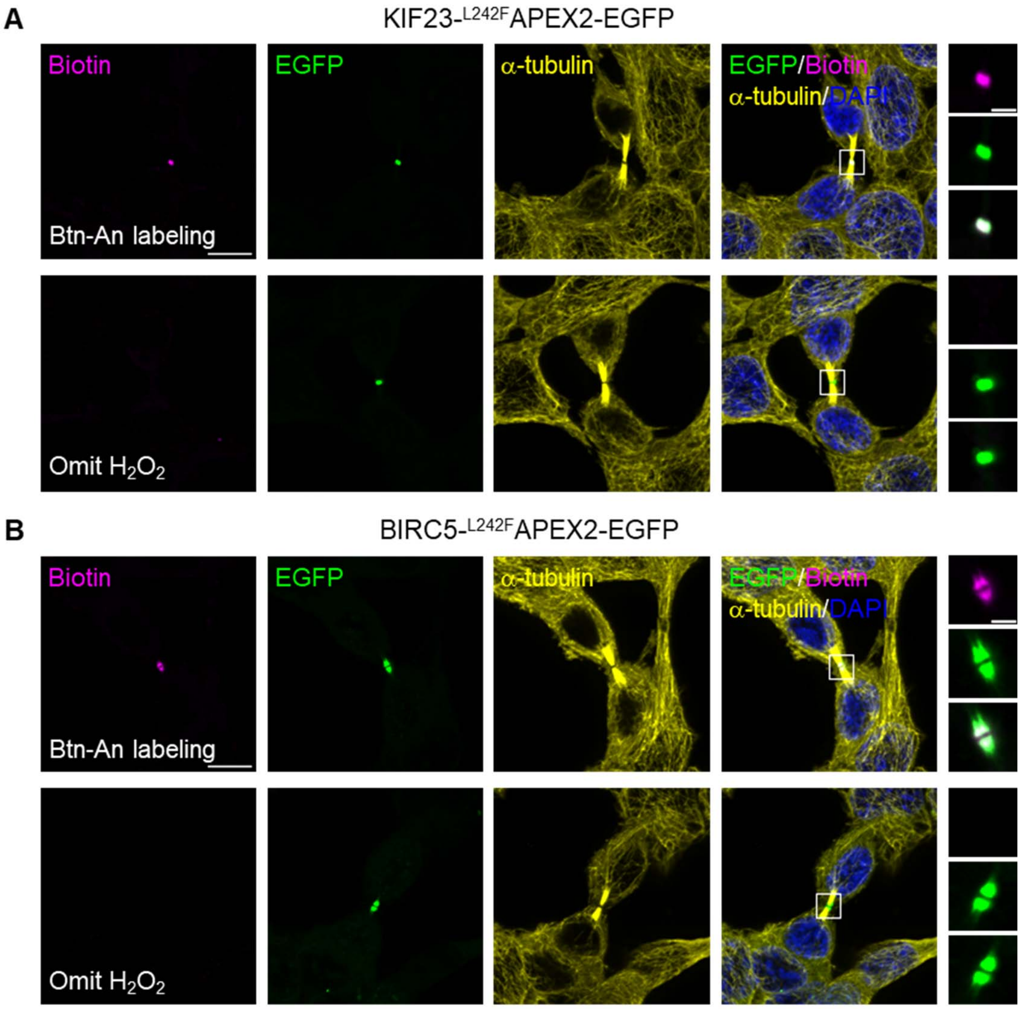

Figure S21. Immunofluorescence imaging of ^{L242F}APEX2-mediated labeling in knock-in cell lines. Representative immunofluorescence images illustrate the distribution of ^{L242F}APEX2 (green), biotinylation (magenta), α-tubulin (yellow) and DAPI-stained nuclei (blue) in cell lines with ^{L242F}APEX2-EGFP knocked in at the *KIF23* (**A**) or *BIRC5* (**B**) locus. For each cell line, images include Btn-An labeled condition (top row), and the negative control omitting H_2O_2 (bottom row). The rightmost panels are magnified views of the boxed regions, displaying the biotinylation channel (top), EGFP channel (middle), and a merged image of both signals (bottom). Scale bars: 10 μm (overview); 2 μm (zoom-in regions).

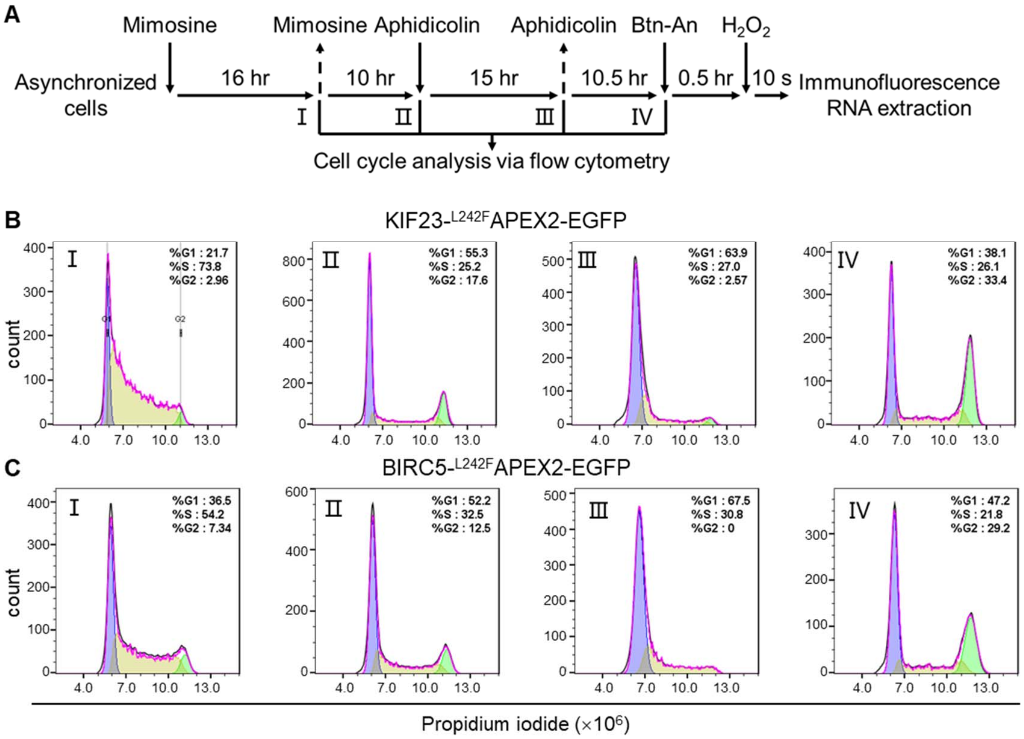

Figure S22. Cell cycle synchronization of endogenously tagged ^{L242F}APEX2-EGFP cell lines. (**A**) Workflow of the synchronization protocol using sequential treatment with mimosine and aphidicolin. Cells were harvested at four time points for flow cytometry analysis: (I) immediately after mimosine block; (II) 10 hours after removal of mimosine, just before aphidicolin block; (III) immediately after aphidicolin block; and (IV) 10.5 hours after release from aphidicolin. (**B**–**C**) Flow cytometry analysis of DNA content (propidium iodide staining) for synchronized KIF23-^{L242F}APEX2-EGFP (**B**) or BIRC5-^{L242F}APEX2-EGFP (**C**) at the stages indicated in (**A**). The percentage of cells in G1, S, and G2/M phases were estimated using FlowJo software.

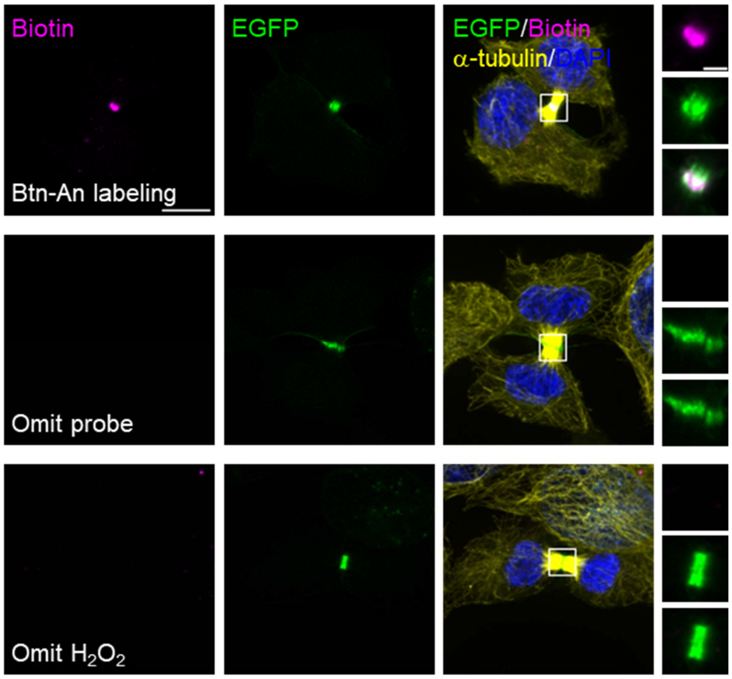

Figure S23. Immunofluorescence analysis of labeling specificity in synchronized BIRC5-^{L242F}APEX2-EGFP cells. Representative immunofluorescence images illustrate the distribution of ^{L242F}APEX2 (green), biotinylation (magenta), α-tubulin (yellow) and DAPI-stained nuclei (blue) in cell cycle-synchronized cells. Rows compare the complete labeling reaction (top) to controls omitting either the Btn-An probe (middle) or H_2O_2 (bottom). The rightmost panels are magnified views of the boxed regions, displaying the biotinylation channel (top), EGFP channel (middle), and a merged image of both signals (bottom). Scale bars: 10 μm (overview); 2 μm (zoom-in regions).

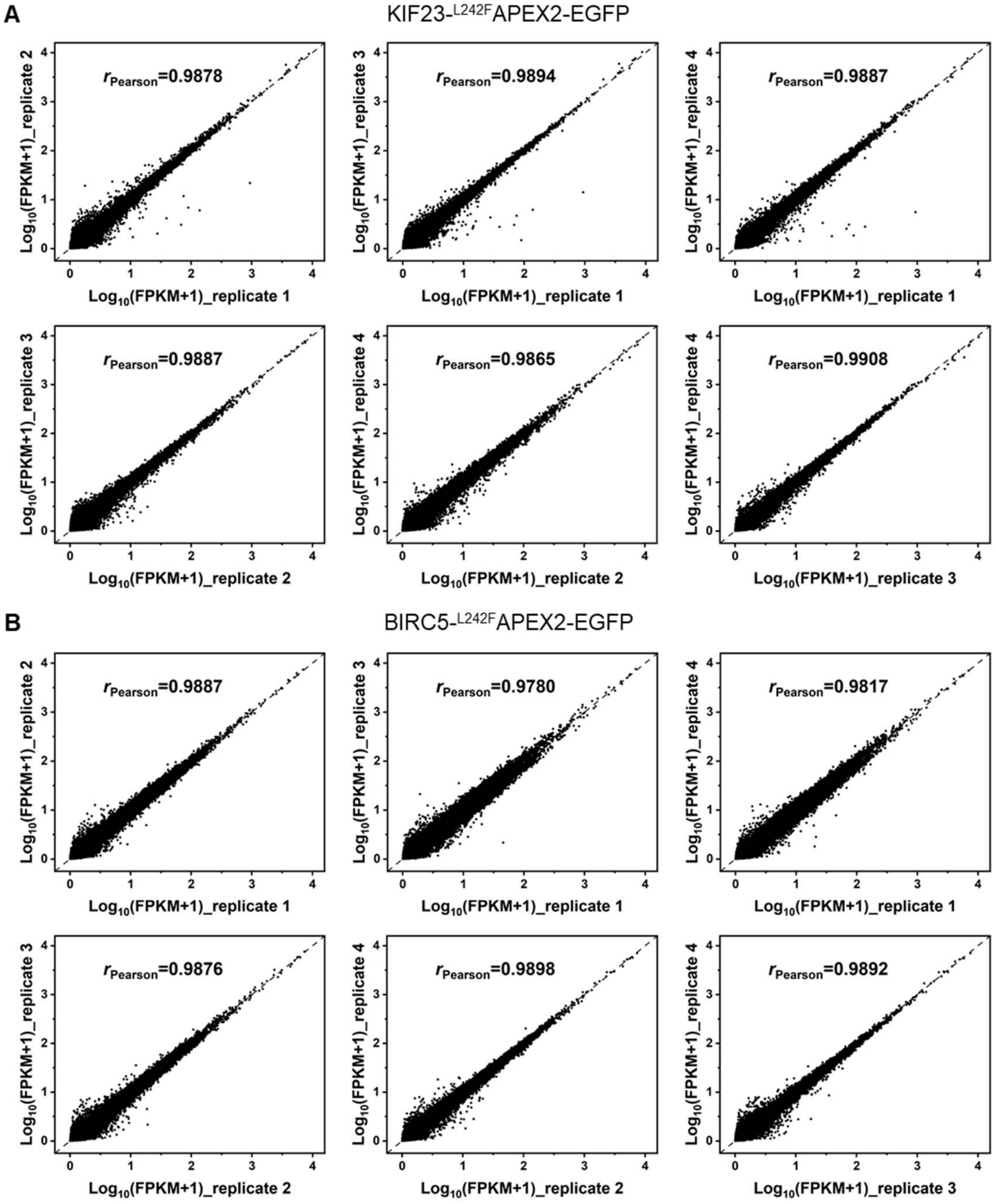

Figure S24. Reproducibility of RNA-seq data across biological replicates. Scatter plots comparing normalized gene expression levels between labeled samples from four independent biological replicates for the KIF23-^{L242F}APEX2-EGFP (**A**) and BIRC5-^{L242F}APEX2-EGFP (**B**) cell lines.

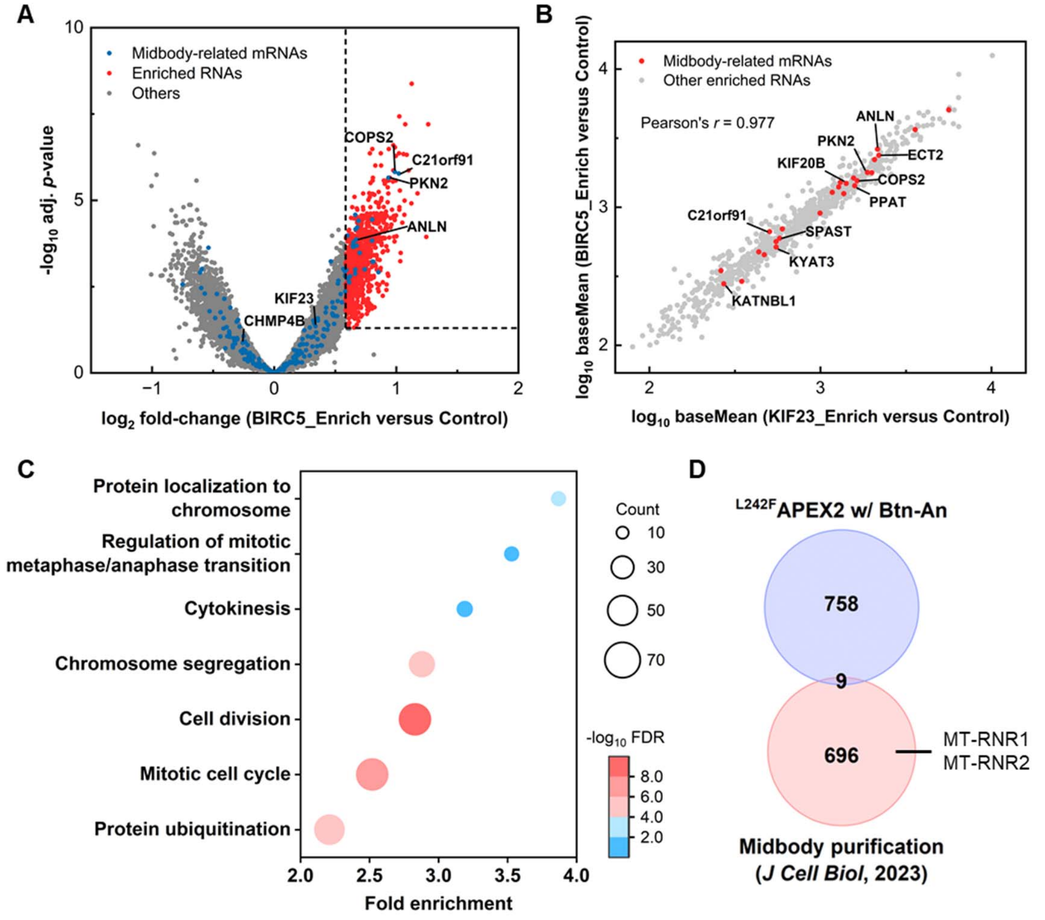

Figure S25. Midbody-proximal transcriptome profiling with ^{L242F}APEX2/Btn-An-mediated labeling. (**A**) Volcano plot of RNAs enriched by BIRC5-^{L242F}APEX2-EGFP. Significantly enriched RNAs are shown in red, and midbody-related mRNAs are highlighted in blue. Dashed lines indicate significance (horizontal, adj. *p*-value = 0.05) and fold-change thresholds (vertical, $\log_2$ fold-change = 0.585). (**B**) Scatter plot showing the concordance of normalized expression estimates for enriched RNAs between two independent DESeq2 analysis protocol. Transcripts chosen for smFISH validation are labeled. (**C**) Bubble plot depicting GO Biological Process (GOBP) enrichment analysis of 763 midbody-proximal mRNAs captured by ^{L242F}APEX2. The circle size represents the number of genes per term, and color indicates the statistical significance. (**D**) Overlap analysis between midbody-localized RNAs identified by ^{L242F}APEX2 labeling and those by biochemical fractionation. Notably, mitochondrial rRNAs *MT-RNR1* and *MT-RNR2* were detected in the fractionation data, suggesting potential mitochondrial contamination.

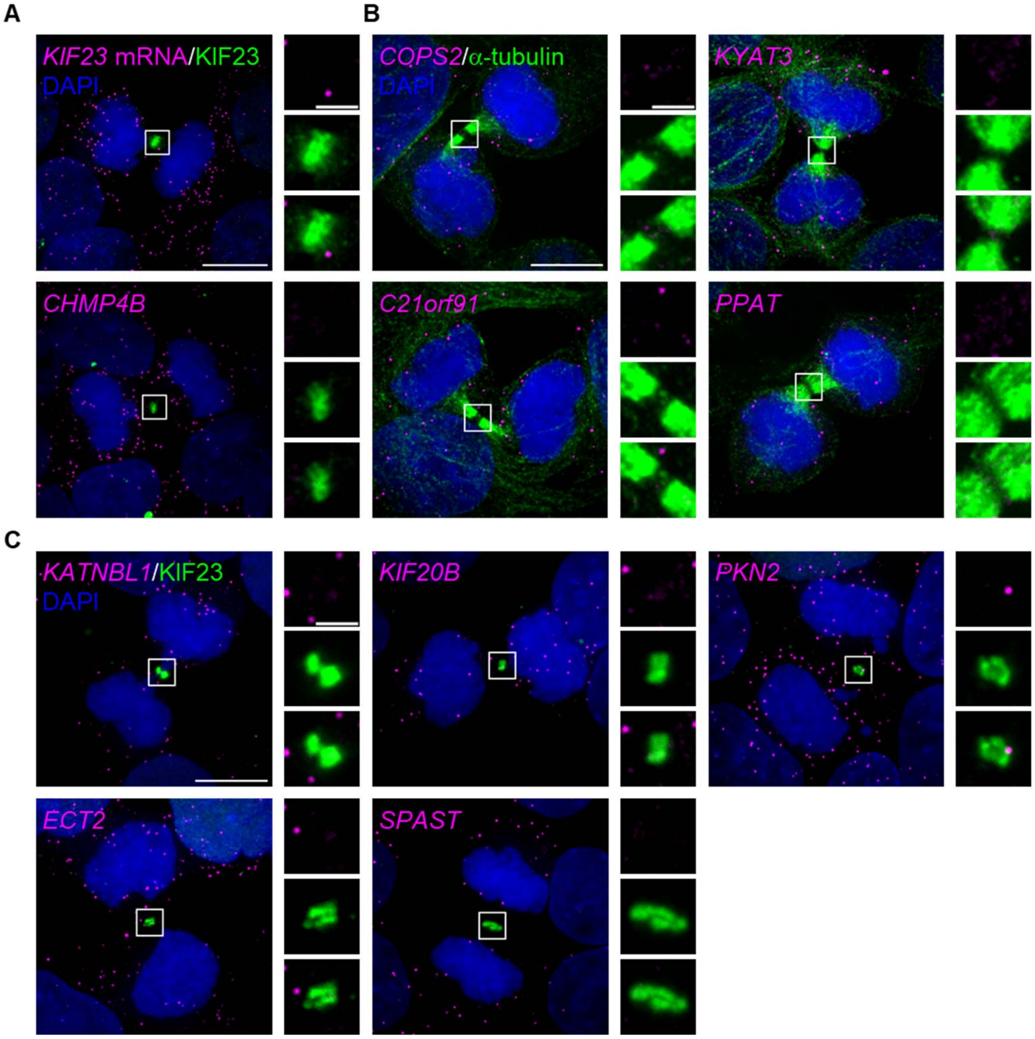

Figure S26. smFISH imaging of candidate RNAs in HEK293T cells during telophase. Representative smFISH images of RNAs identified by prior biochemical fractionation (**A**) or ^{L242F}APEX2 labeling (**B**–**C**). Magenta: endogenous mRNA; green: anti-KIF23 to stain the midbody (**A** and **C**), or anti-α-tubulin to visualize the microtubules (**B**); blue: DNA stained with DAPI. For each target, magnified views of boxed regions show the RNA channel (top), protein marker channel (middle), and a merged image (bottom). Scale bars: 10 µm (overview); 2 µm (zoom-in regions).

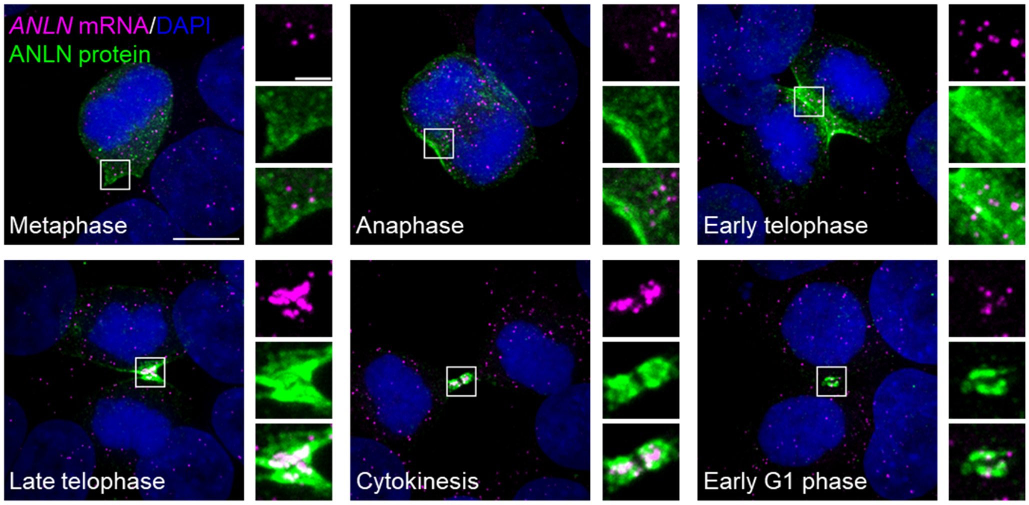

Figure S27. Co-localization analysis of ANLN mRNA and protein during mitosis. Simultaneous detection of *ANLN* mRNA (by smFISH, magenta) and ANLN protein (by immunofluorescence, green) in fixed cells at indicated mitotic stages. DNA are visualized with DAPI (blue). For each phase, magnified views of boxed regions show the mRNA signal (top), protein signal (middle), and a merged image of both signals (bottom). Scale bars: 10 μm (overview); 2 μm (zoom-in regions).

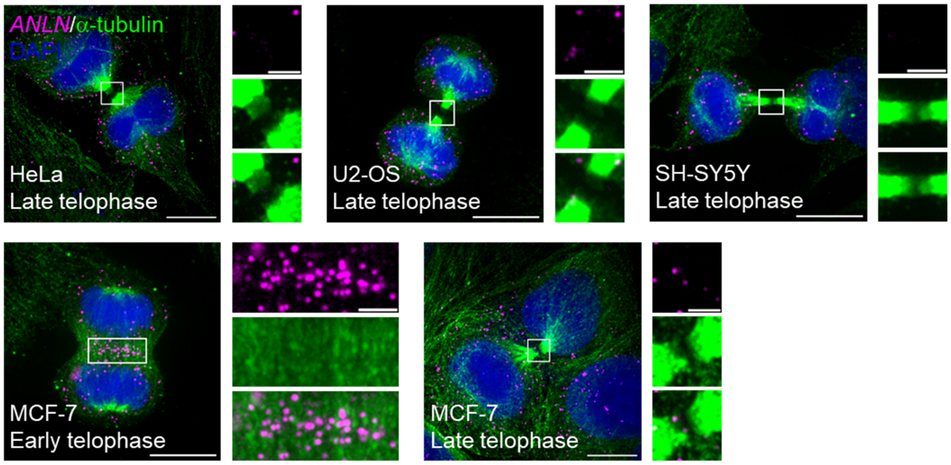

Figure S28. Localization of *ANLN* mRNA across different cell types during telophase. Representative smFISH images illustrate the distribution of *ANLN* mRNA (magenta) in the indicated cell lines. Microtubules are labeled with anti-α-tubulin (green), and DNA is stained with DAPI (blue). For each cell line, magnified views of boxed regions show the RNA channel (top), protein marker channel (middle), and a merged image (bottom). Scale bars: 10 μm (overview); 2 μm (zoom-in regions).

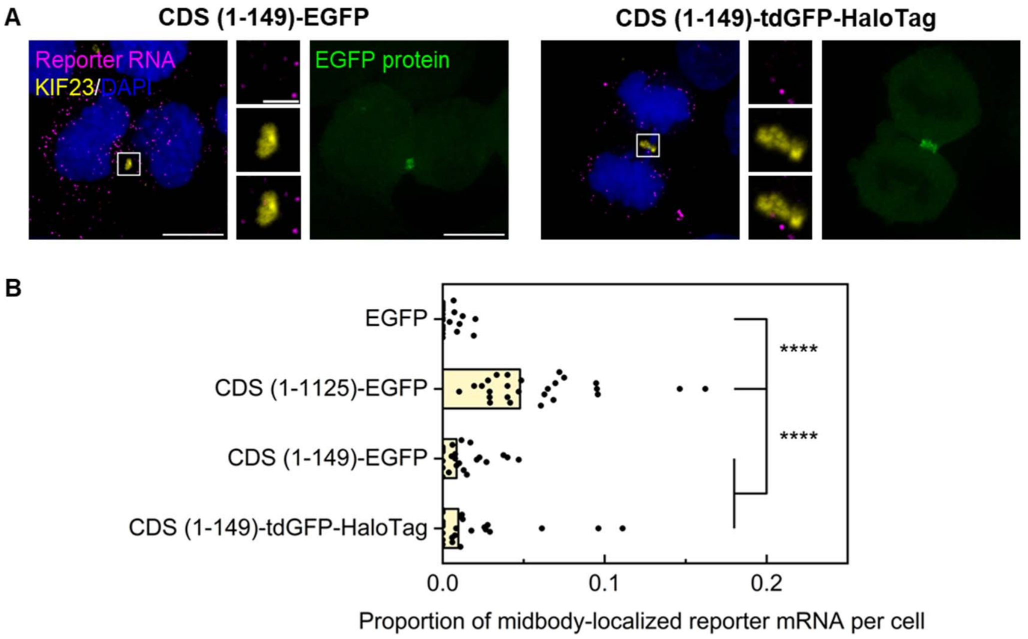

Figure S29. Localization of EGFP reporter mRNA during telophase. (**A**) Simultaneous detection of indicated EGFP reporter mRNA (by smFISH, magenta) and its corresponding EGFP fusion protein (green). The midbody is labeled with anti-KIF23 (yellow), and DNA is stained with DAPI (blue). For each construct, magnified views of boxed regions show the RNA channel (top), the midbody marker channel (middle), and a merged image (bottom). Scale bars: 10 μm (overview); 2 μm (zoom-in regions). (**B**) Quantification of reporter mRNA localization at the midbody. Bar plot shows the proportion of midbody-localized reporter mRNA per cell, presented as median ($n = 21$, 26, 23, and 24 cells from two independent experiments). Statistical significance was calculated by a two-sided Mann-Whitney test. ****: $p < 0.0001$. The control EGFP reporter exhibits a median colocalization percentage of 0%, indicating that over half of cells showed no detectable signal at the midbody.

Supplementary Tables

Table S1. Key resources table used in this study

REAGENT or RESOURCE	SOURCE	IDENTIFIER
**Antibodies**		
V5-Tag Monoclonal antibody (3C8)	Biodragon	Cat# B1005
α-tubulin Monoclonal antibody (BF11)	Biodragon	Cat# B1052
Rabbit anti-HSP60 antibody	abcam	Cat# ab46798
Rabbit anti-Calnexin antibody	abcam	Cat# ab22595
Rabbit Recombinant Monoclonal MKLP1 antibody	abcam	Cat# ab174304
Rat alpha Tubulin Monoclonal Antibody (YL1/2)	Invitrogen	Cat# MA180017
Survivin (71G4B7) Rabbit Monoclonal Antibody	Cell Signaling Technology	Cat# 2808
Anillin (F6M2V) Rabbit Monoclonal Antibody	Cell Signaling Technology	Cat# 48298
HRP-Goat anti-Mouse IgG (H+L)	Biodragon	Cat# BF03001
HRP-Goat anti-Rabbit IgG (H+L)	Biodragon	Cat# BF03008
Streptavidin, Alexa Fluor 568 conjugate	Invitrogen	Cat# S11226
Goat anti-Mouse IgG (H+L) Highly Cross-Adsorbed Secondary Antibody, Alexa Fluor 647	Invitrogen	Cat# A-21236
Goat anti-Rat IgG (H+L) Cross-Adsorbed Secondary Antibody, Alexa Fluor 488	Invitrogen	Cat# A-11006
Goat anti-Rabbit IgG (H+L) Highly Cross-Adsorbed Secondary Antibody, Alexa Fluor 568	Invitrogen	Cat# A-11036

Chemicals, peptides, and recombinant proteins		
D-(+)-glucose	Sigma	Cat# G6152-500G
Yeast Nitrogen Base, without Amino Acid	BD	Cat# 291940
Yeast Synthetic Drop-out Medium Supplements – without tryptophan	Sigma-Aldrich	Cat# Y1876
Yeast Extract	OXOID	Cat# LP0042
Peptone	Amresco	Cat# J636
Ampicillin	Inalco	Cat# 1758-9314
Dulbecco's Modified Eagle's Medium	Gibco	Cat# C11995500BT
McCoy's 5A Medium	VivaCell	C3020-0500
0.05% Trypsin-EDTA	Gibco	Cat# 25300120
Fetal bovine serum	Gibco	Cat# 10099141C
Opti-MEM I Reduced Serum Medium	Gibco	Cat# 31985062
Lipofectamine 3000 Transfection Reagent	Invitrogen	Cat# L3000015
Matrigel Matrix	Corning	Cat# 356234
TRIzol reagent	Invitrogen	Cat# 15596018
DNase I (RNase-free)	NEB	Cat# M0303
Yeast tRNA	Invitrogen	Cat# 15401011
Bovine serum albumin	Sangon Biotech	Cat# A500023-0100
Dynabeads MyOne Streptavidin C1 beads	Invitrogen	Cat# 65002
RiboLock RNase Inhibitor	Thermo Scientific	Cat# EO0384
NaCl (5 M), RNase-free	Invitrogen	Cat# AM9759
EDTA (0.5 M), pH 8.0, RNase-free	Invitrogen	Cat# AM9260G

UltraPure 1 M Tris-HCl Buffer, pH 7.5	Invitrogen	Cat# 15567027
PBS (10×) pH 7.4, RNase-free	Invitrogen	Cat# AM9625
D-Biotin Solution (50 mM)	Invitrogen	Cat# B20656
Lithium acetate	Energy Chemical	Cat# E060045
DMSO	Aladdin	Cat# D103273
Formamide	Sigma-Aldrich	Cat# F9037
Urea	Sigma-Aldrich	Cat# U5378
Sodium hydroxide solution	Sigma-Aldrich	Cat# S2770
Tween-20	Sigma-Aldrich	Cat# P1379
SDS, 10% Solution, RNase-free	Invitrogen	Cat# AM9822
BeyoPure Ultrapure Water	Beyotime	Cat# ST876
Glycogen, RNA grade	Thermo Scientific	Cat# R0551
Puromycin	J&K	Cat# 168086
Harringtonine	TargetMol	Cat# T8286
Cycloheximide	Fluorochem	Cat# 375034
Leptomycin B	Beyotime	Cat# S1726
Mimosine	MedChemExpress	Cat# HY-N0928
Aphidicolin	MedChemExpress	Cat# HY-N6733
Propidium iodide	Sigma-Aldrich	Cat# P4170
RNase A	Thermo Scientific	Cat# R1253

Hydrogen Preoxide-30%	XiLONG	Cat# S6364
Sodium ascorbate	Aladdin	Cat# S105024
Trolox	Sigma-Aldrich	Cat# 238813
cOmplete, EDTA-free, EASYpack	Roche	Cat# 04693132001
Biotin-phenol (Btn-Ph)	Reference [1]	PMID: 31240809
Biotin-aniline (Btn-An)	Reference [1]	PMID: 31240809
Biotin-naphthylamine (Btn-Nap)	Reference [1]	PMID: 31240809
BP5	Reference [2]	PMID: 33397984
SSC (20×), RNase-free	Invitrogen	Cat# AM9770
tRNA from *E. coli* MRE 600	Roche	Cat# 10109541001
UltraPure Salmon Sperm DNA Solution	Invitrogen	Cat# 15632011
Ribonucleoside Vanadyl Complex (RVC)	NEB	Cat# S1402S
Dextran sulfate sodium salt	Sigma-Aldrich	Cat# D6001
Paraformaldehyde	Sigma-Aldrich	Cat# V900894
$MgCl_2$ (1 M)	Invitrogen	Cat# AM9530G
Glycine	Solarbio	Cat# G8200
Fluoromount-G Anti-Fade	SouthernBiotech	Cat# 0100-35
PowerUp SYBR Green Master Mix	Applied Biosystems	Cat# A25742
EndoFree Mini PlasmidKit II	TIANGEN	Cat# DP118-02

Gel Extraction Kit	Omega	Cat# D2500-02
Frozen-EZ Yeast Transformation II Kit	Zymo research	Cat# T2001
Lightening Cloning Kit	Biodragon	Cat# BDIT0014
Phanta Max Super-Fidelity DNA Polymerase	Vazyme	Cat# P505
2×Pfu MasterMix	CWBIO	Cat# CW0686A
8-oxo-dGTP	Enzo	Cat# JBS-NU-1117S
dPTP	Enzo	Cat# JBS-NU-1119S
Taq DNA Polymerase with Standard *Taq*(Mg-free) Buffer	NEB	Cat# M0320S
NheI-HF	NEB	Cat# R3131S
BamHI-HF	NEB	Cat# R3136S
BsmBI-v2	NEB	Cat# R0739S
VAHTS DNA Clean Beads	Vazyme	Cat# N411
DNA Clean & Concentrator-5	Zymo Research	Cat# D4014
RNA Clean & Concentrator-25	Zymo Research	Cat# R1018
**Critical commercial assays**		
CellTiter 96 AQ_{ueous} One Solution Cell Proliferation Assay	Promega	Cat# G3580
NEBNext Ultra II RNA Library Prep Kit for Illumina	NEB	Cat# E7770
NEBNext Poly(A) mRNA Magnetic Isolation Module	NEB	Cat# E7490L
Fragment Analyzer DNA/NGS Kits	AATI	Cat# DNF-474-0500
ProtoScript II First Strand cDNA Synthesis Kit	NEB	Cat# E6560

**Experimental models: Cell lines**		
HEK293T	National Infrastructure of Cell Line Resource	1101HUM-PUMC000212
HeLa S3	National Infrastructure of Cell Line Resource	1101HUM-PUMC000188
SH-SY5Y	National Infrastructure of Cell Line Resource	1101HUM-PUMC000026
U-2 OS	National Infrastructure of Cell Line Resource	1101HUM-PUMC000028
MCF-7	Laboratory of Prof. Prof. Mo Li, Peking University	N/A
**Oligonucleotides**		
qPCR primers	This study	Table S2
CRISPR/Cas9 gRNA	This study	Table S4
Genotyping primers	This study	Table S4
smFISH probes	This study	Data S3
**Recombined DNA**		
pCTcon2-Aga2p-V5-APEX2	This study	-
pCTcon2-Aga2p-V5- ^{L242F}APEX2	This study	-
pCTcon2-Aga2p-V5- ^{L242W}APEX2	This study	-
pCTcon2-Aga2p-V5- ^{L242Y}APEX2	This study	-
pCTcon2-Aga2p-V5- ^{L242H}APEX2	This study	-
pCTcon2-Aga2p-V5- ^{L242P}APEX2	This study	-
pLX304-V5-^{L242F}APEX2-NES-EGFP	This study	-

pLX304-mito-V5-^{L242F}APEX2	This study	-
pLX304-V5- ^{L242F}APEX2-Sec61b	This study	-
pLX304-V5-EGFP	This study	-
pLX304-V5-APEX2-EGFP	This study	-
pLX304-V5-^{L242F}APEX2-EGFP-3×NLS	This study	-
lentiCRISPRv2	Prof. Mo Li, Peking University	Addgene# 98290
EGFP reporter plasmids	This study	Table S5
**Software and algorithms**		
HISAT2	Reference [3]	RRID: SCR_015530
HTSeq	Reference [4]	RRID: SCR_005514
DESeq2	Reference [5]	RRID: SCR_015687
Fiji	Reference [6]	RRID: SCR_002285
PANTHER	Reference [7]	RRID: SCR_004869
μManager	Reference [8]	RRID: SCR_000415
Cellpose3	Reference [9]	PMID: 39939718
EzColocalization	Reference [10]	PMID: 30361629
Oligostan	Reference [11]	PMID: 27599845
RS-FISH	Reference [12]	PMID: 36396787

Table S2. qPCR primers used in this study

Primer name	Sequence
MT-CYB_qPCR_F	TCGGAGGACAACCAGTAAGC
MT-CYB_qPCR_R	GTTTTCAATTAGGGAGATAGTTGGT
MT-CO2_qPCR_F	AACCAAACCACTTTCACCGC
MT-CO2_qPCR_R	CGATGGGCATGAAACTGTGG
MT-ND1_qPCR_F	CACCTCTAGCCTAGCCGTTT
MT-ND1_qPCR_R	CCGATCAGGGCGTAGTTTGA
TMX1_qPCR_F	ACGGACGAGAACTGGAGAGA
TMX1_qPCR_R	ATTTTGACAAGCAGGGCACC
CANX_qPCR_F	GGTGCTTGGAACTGCTATTG
CANX_qPCR_R	CCCTGTTGGAACTGGAGCTT
SSR2_qPCR_F	GTTTGGGATGCCAACGATGAG
SSR2_qPCR_R	CTCCACGGCGTATCTGTTCA
GAPDH_qPCR_F	TTCGACAGTCAGCCGCATCTTCTT
GAPDH_qPCR_R	GCCCAATACGACCAAATCCGTTGA
XIST_qPCR_F	CCCTACTAGCTCCTCGGACA
XIST_qPCR_R	ACACATGCAGCGTGGTATCT
APEX2_qPCR_F	AGGGAACCAAAACAGGCGGA
APEX2_qPCR_R	TTCAGAGGTTCCAGCAGCCG
BIRC5_qPCR_F	ATTTGATTCGCCCTCCTCCC
BIRC5_qPCR_R	TCCAGAGGTTTCCAGCGAAG
KIF23_qPCR_F	TACCCATTTGAATCGTGAGTCCA
KIF23_qPCR_R	CTCTGGTCCGGTTAGTTCTTTC

Table S3. List of GO terms used to define GOCC-secretome

GO term	GO number
Plasma membrane	GO:0005886
Membrane	GO:0016020
Extracellular region	GO:0005576
Extracellular space	GO:0005615
Endoplasmic reticulum	GO:0005783
Endoplasmic reticulum membrane	GO:0005789
Endoplasmic reticulum lumen	GO:0005788
Signal recognition particle, endoplasmic reticulum targeting	GO:0005786
Golgi apparatus	GO:0005794
Golgi membrane	GO:0000139
Golgi lumen	GO:0005796
Endosome	GO:0005768
Endosome membrane	GO:0010008
Lysosome	GO:0005764
Lysosomal membrane	GO:0005765
Cytoplasmic vesicle	GO:0031410
Peroxisome	GO:0005777
Vesicle	GO:0031982
ER to Golgi transport vesicle membrane	GO:0012507

Table S4. gRNA sequences and genotyping primers

Name	Sequence
KIF23_gRNA_forward	CAAAGTTCAACTCTAAGTGC
KIF23_gRNA_reverse	TGAAAATGTTCTTTCAGTAC
KIF23_genotyping_F1	GCTCATGTTCATATTCTGCCAACGC
KIF23_genotyping_R1	AACAATGAGTGCCAGGTAAATGCAG
BIRC5_gRNA_forward	CTGCCATGGATTGAGGCCTC
BIRC5_gRNA_reverse	CGGCCAGAGGCCTCAATCCA
BIRC5_genotyping_F2	GAAAGGTAGGGCAGTGGTTAAGAGC
BIRC5_genotyping_R2	TTAAACAGTAGAGGAGCCAGGGACT

Table S5. EGFP reporter plasmids used in this study

Reporter name	Description
EGFP	pUbC-EGFP
EGFP-ANLN 3'UTR	pUbC-EGFP-ANLN 3'UTR
ANLN 5'UTR-EGFP	pUbC-ANLN 5'UTR-EGFP
ANLN CDS-EGFP	pUbC-ANLN CDS-EGFP
CDS (Δ2-149)	pUbC-ANLN CDS (Δ2-149 aa)-EGFP
CDS (Δ150-250)	pUbC-ANLN CDS (Δ150-250 aa)-EGFP
CDS (Δ251-455)	pUbC-ANLN CDS (Δ251-455 aa)-EGFP
CDS (Δ456-645)	pUbC-ANLN CDS (Δ456-645 aa)-EGFP
CDS (Δ646-981)	pUbC-ANLN CDS (Δ646-981 aa)-EGFP
CDS (Δ982-1125)	pUbC-ANLN CDS (Δ982-1125 aa)-EGFP
CDS (1-149)-EGFP	pUbC-ANLN CDS (1-149 aa)-EGFP
CDS (1-149)-tdGFP-HaloTag	pUbC-ANLN CDS (1-149 aa)-tdGFP-HaloTag

References

[1] Zhou, Y.; Wang, G.; Wang, P.; Li, Z.; Yue, T.; Wang, J.; Zou, P., Expanding APEX2 substrates for proximity-dependent labeling of nucleic acids and proteins in living cells. *Angew. Chem. Int. Ed.*, **2019**, 58 (34), 11763-11767.

[2] Ke, M.; Yuan, X.; He, A.; Yu, P. Y.; Chen, W. D.; Shi, Y.; Hunter, T.; Zou, P.; Tian, R. J., Spatiotemporal profiling of cytosolic signaling complexes in living cells by selective proximity proteomics. *Nat. Commun.*, **2021**, 12 (1), 71.

[3] Kim, D.; Langmead, B.; Salzberg, S. L., HISAT: a fast spliced aligner with low memory requirements. *Nat. Methods*, **2015**, 12 (4), 357-360.

[4] Anders, S.; Pyl, P. T.; Huber, W., HTSeq--a Python framework to work with high-throughput sequencing data. *Bioinformatics*, **2015**, 31 (2), 166-169.

[5] Love, M. I.; Huber, W.; Anders, S., Moderated estimation of fold change and dispersion for RNA-seq data with DESeq2. *Genome Biol.*, **2014**, 15 (12), 550.

[6] Schindelin, J.; Arganda-Carreras, I.; Frise, E.; Kaynig, V.; Longair, M.; Pietzsch, T.; Preibisch, S.; Rueden, C.; Saalfeld, S.; Schmid, B.; Tinevez, J. Y.; White, D. J.; Hartenstein, V.; Eliceiri, K.; Tomancak, P.; Cardona, A., Fiji: an open-source platform for biological-image analysis. *Nat. Methods*, **2012**, 9 (7), 676-682.

[7] Thomas, P. D.; Ebert, D.; Muruganujan, A.; Mushayahama, T.; Albou, L. P.; Mi, H., PANTHER: Making genome-scale phylogenetics accessible to all. *Protein Sci.*, **2022**, 31 (1), 8-22.

[8] Edelstein, A. D.; Tsuchida, M. A.; Amodaj, N.; Pinkard, H.; Vale, R. D.; Stuurman, N., Advanced methods of microscope control using μManager software. *J Biol Methods*, **2014**, 1 (2), e10.

[9] Stringer, C.; Pachitariu, M., Cellpose3: one-click image restoration for improved cellular segmentation. *Nat Methods*, **2025**, 22 (3), 592-599.

[10] Stauffer, W.; Sheng, H.; Lim, H. N., EzColocalization: An ImageJ plugin for visualizing and measuring colocalization in cells and organisms. *Sci Rep*, **2018**, 8 (1), 15764.

[11] Tsanov, N.; Samacoits, A.; Chouaib, R.; Traboulsi, A. M.; Gostan, T.; Weber, C.; Zimmer, C.; Zibara, K.; Walter, T.; Peter, M.; Bertrand, E.; Mueller, F., smiFISH and FISH-quant - a flexible single RNA detection approach with super-resolution capability. *Nucleic Acids Res*, **2016**, 44 (22), e165.

[12] Bahry, E.; Breimann, L.; Zouinkhi, M.; Epstein, L.; Kolyvanov, K.; Mamrak, N.; King, B.; Long, X.; Harrington, K. I. S.; Lionnet, T.; Preibisch, S., RS-FISH: precise, interactive, fast, and scalable FISH spot detection. *Nat Methods*, **2022**, 19 (12), 1563-1567.
